# Molecular Allergen Sensitization Profiling in a Large Cohort of Dogs Suspected of Allergic Diseases in the USA: Prevalence and Unsupervised IgE Clustering (2024–2025)

**DOI:** 10.3390/ani16152414

**Published:** 2026-08-05

**Authors:** Thierry Olivry, Ana Mas-Fontao

**Affiliations:** 1 Nextmune AB, Riddargatan 17, 114 57 Stockholm, Sweden; 2 Nextmune Spain, Valentin Beato 24, 28037 Madrid, Spain

**Keywords:** allergens, allergy, dogs, IgE, molecular allergology, PAX, Pet Allergy Xplorer, serology, USA

## Abstract

Allergen-specific IgE multiplex serology enables the molecular characterization of sensitizations in large populations. To determine the sensitization prevalence and spectrum in dogs suspected of having allergies in the US, we analyzed results from 21,121 serum samples tested in 2024 and 2025 using the Pet Allergy Xplorer (PAX) 238-allergen platform. Sensitization to at least one allergen and to at least one environmental allergen was detected in 90.7% and 86.2% of dogs, respectively. House dust mites (HDMs) were the predominant allergens (79.5%), followed by Hymenoptera venoms (39.8%), meats (21.9%), weeds (21.5%), and tree pollens (18.6%). Sensitization to the *Dermatophagoides farinae* HDM was the most prevalent (76.7%). Unsupervised clustering of 16,366 sensitized dogs identified six biologically coherent profiles centered on flea saliva, mammalian serum albumins/IgGs, honeybee venom, HDM group-2 allergens, storage mites, and PR-10-family allergens. A second, pollen/plant-food-focused analysis found clusters associated with *Parietaria* Par j 2, ragweed, granule-bound starch synthases, and PR-10 allergens, as well as a minor Phl p 6-associated grass pollen profile. Altogether, these results define the molecular sensitization repertoire of dogs suspected of allergic diseases in the US and provide the background for interpreting IgE serological results and optimizing allergen selection for immunotherapy.

## Simple Summary

Dogs with environmental allergies often have blood tests to identify allergens that may contribute to their disease and to guide allergen immunotherapy (desensitization). In this study, we analyzed results from more than 21,000 allergy blood tests performed on dogs suspected of having allergies across the United States in 2024 and 2025 using the PAX test, which measures allergy-related antibody levels for 238 allergens. Most dogs reacted to at least one environmental allergen. House dust mites were by far the most common allergens detected, followed by insect venoms, meat proteins, weeds, and tree pollens. Using computer-based analyses, we also identified several recurring patterns of allergen sensitization, including reactions dominated by flea saliva, dust mites, storage mites, honeybee venom, animal proteins, and specific pollen and plant-food allergens. This is the first large-scale, molecular-level characterization of allergen sensitization in dogs in the United States. These observations improve our knowledge of canine allergies at the population level and may help veterinarians interpret allergy test results more accurately and select allergens for immunotherapy more effectively.

## 1. Introduction

In the United States, allergic skin diseases account for 3–4% of diagnoses, making them the second most common disorder after dental calculus, otitis externa, and gingivitis [1]. Although this prevalence appears low, several health surveys suggest that it has been increasing over the last few decades [2]. After diagnosing allergic disease in dogs and in preparation for allergen immunotherapy, veterinarians often perform intradermal (IDT) and/or IgE serological tests (ISTs) to identify allergen sensitizations that may trigger allergic symptoms [3]. Aggregating test results from the same testing method into large-scale datasets enables the characterization of allergen sensitization at the population level. Such analyses can help identify the most prevalent allergen categories and pinpoint common sensitization patterns. Furthermore, as shown recently in human allergic patients [4], unsupervised machine learning approaches can identify molecular sensitization profiles that might not be apparent from conventional prevalence analyses.

The interpretation of historical sensitization surveys is complicated by substantial methodological heterogeneity. As highlighted in a recent systematic review [5], most studies included relatively small numbers of dogs and relied on different testing platforms using allergen extracts that differ considerably in composition [6]; consequently, direct comparison of sensitization profiles among studies is notoriously difficult.

To date, only a handful of large surveys involving more than 1000 allergic dogs have been reported, including studies from Australia, Norway, France, Scandinavia, and Europe [7,8,9,10,11].

Despite the large canine population and the widespread use of IDT and IST in the United States, we identified only two small surveys reporting allergen-specific sensitization data determined by IDT in more than 100 American dogs: Hillier et al. 2000 (mite extracts only; 115 dogs) [12] and Mueller et al. 2002 (268 dogs) [13]. Because environmental exposure is a major determinant of IgE sensitization, differences in climate, flora, insect and arthropod fauna, and regional allergen distribution between Australia, Europe and the United States are likely to result in distinct molecular sensitization patterns.

In this paper, we report the sensitization profiles of 21,121 American dogs suspected of allergic diseases, which were tested in 2024 and 2025 using the Pet Allergy Xplorer (PAX) multiplex molecular allergen macroarray (Nextmune, Stockholm, Sweden), a platform that includes one-third of traditional allergen extracts and two-thirds of molecular allergens [11]. Unlike traditional allergen extracts, molecular allergology enables the identification of sensitization to individual allergenic molecules rather than complex and often heterogeneous allergen mixtures. This improved resolution facilitates the recognition of biologically coherent sensitization patterns, as well as the identification of primary sensitizations and potential cross-reactivities that are difficult to discern using extract-based testing alone [14].

Accordingly, the objectives of this study were to characterize the prevalence of allergen-specific IgE sensitization in American dogs tested for suspected allergic disease, identify recurring molecular sensitization profiles using unsupervised machine learning approaches, and establish a reference framework for comparison with other canine populations, including European dogs tested with the same platform [11].

## 2. Materials and Methods

### 2.1. PAX Test Results

This retrospective laboratory database study analyzed a consecutive convenience sample of 32,710 sera from dogs with suspected allergic disease submitted to the Nextmune US laboratory (Phoenix, AZ, USA) for routine PAX allergen-specific IgE testing between 1 January 2024, and 31 December 2025; as such, the study population does not represent a random sample of the general US canine population. Because this was a retrospective analysis of routine diagnostic submissions, no clinical inclusion or exclusion criteria for concurrent diseases, including ectoparasitic infestations or infections, were applied. Finally, there was no standardized information on age, breed, sex, geographic origin within the United States, clinical diagnosis, medication history, or disease severity accompanying the laboratory submissions, so these parameters were unavailable for analysis. 

From the initial set of test results, we removed those of 5146 dogs with detectable IgE against cross-reactive carbohydrate determinants (CCDs) (i.e., specific IgE [sIgE] levels of 28 ng/mL or higher against one or both “CCD detectors”) after the PAX’s routine CCD-IgE blocking [11]. These samples were excluded because CCD-IgE can lead to false-positive detection of polysensitization to pollen and plant food extracts, as well as to native plant components, in serological assays [15]. From the remaining 27,564 dogs, we removed results for 6443 dogs from other countries, leaving a dataset of 21,121 dogs (2024: 10,261 dogs; 2025: 10,860) living in the USA. Each laboratory submission was treated as an independent sample. Because the dataset was anonymized and contained only laboratory accession numbers, it was not possible to determine whether more than one sample originated from the same dog. Consequently, repeat submissions from a small number of dogs cannot be excluded, although these are unlikely to have materially influenced the analyses given the size of the study population.

The PAX cartridges are used with different secondary monoclonal antibodies for dogs, cats, and horses, and some allergens are reported in one species but not another [11]. The 24.1 version of PAX cartridges contains 274 spotted allergens: 82 extracts and 192 components. For dogs in the USA, specific IgE levels are reported for 238 allergens from 17 biological categories; there are 75 extracts, 2 extract mixes (Jun a_Jun v; Rum a_Rum c), 160 components, and one component mix (Gad m 2 + 3) (Supplementary Table S1); overall, molecular components represent 67.6% of this allergen macroarray.

### 2.2. Data Extraction

#### 2.2.1. Sensitization Prevalence

To report the prevalence of sensitizations, we classified a sample as positive for a given allergen when its measured sIgE concentration was at least 28 ng/mL, consistent with the validated positivity threshold for the PAX [11]. We then determined seropositivity rates across all 17 categories of environmental and food allergens, considering a dog sensitized if sIgE for at least one allergen in that category met or exceeded the threshold.

The dataset contained no missing values because each laboratory submission generated allergen-specific IgE measurements for all allergens included in the PAX testing panel; consequently, no imputation or exclusion of observations due to missing data was required.

#### 2.2.2. Unsupervised Clustering of Sensitizations

##### Clustering of Sensitizations to the Full Allergen Panel

Because most dogs in this cohort were sensitized to the *Dermatophagoides farinae* (Der f) house dust mite (HDM) extract (76.7%), this allergen was excluded to prevent this highly prevalent sensitization from dominating and preventing the formation of relevant clusters. Although sensitization to *Dermatophagoides pteronyssinus* (Der p) was less common, its molecular components largely recapitulated the extract signal, providing limited additional discriminatory information and warranting its exclusion. Sensitization data for both Der f and Der p were nevertheless shown in the heatmap as a reference.

Raw allergen-specific IgE values (ng/mL) were normalized within each dog by dividing each allergen value by the total sIgE signal across the allergen panel, scaling by 1000, and applying a log1p transformation. The 80 most variable allergens among sensitized dogs were selected for downstream analysis to retain the principal sources of variance while limiting the inclusion of low-information features. These variables were standardized (z-score), and principal component analysis (PCA) was performed. The first 15 principal components were used for clustering and uniform manifold approximation and projection (UMAP) visualization.

K-means clustering was initially explored with positivity thresholds of 28, 30, 32, and 35 ng/mL and cluster numbers from k = 2 to 20. Silhouette scores, Davies–Bouldin indices, and inertia curves were computed for each combination. Because the indices were broadly similar across k = 8–14, k = 10 at 30 ng/mL was selected as the smallest value at which rare biologically distinct sensitization profiles were consistently resolved as separate clusters across repeated runs. While the 28 ng/mL threshold used for prevalence analyses reflects the validated clinical positivity cutoff for the diagnostic platform, the 30 ng/mL threshold used herein was chosen based on cluster stability metrics.

Clusters were labeled C1–C10 in descending order of size. For each allergen within each cluster, fold enrichment was calculated as the ratio of the cluster mean of log10(sIgE + 1) to the corresponding mean across all other clusters. Allergens with an arbitrary fold enrichment of 1.20 or higher were designated major cluster markers, whereas those with fold enrichment between 1.10 and 1.19 were considered supporting markers. These thresholds served as effect-size filters appropriate for this large sample size; post hoc Mann–Whitney U testing with Benjamini–Hochberg correction confirmed that all classified markers were statistically significant (BH-FDR, *p* < 0.05).

Heatmaps were generated using log10-transformed raw sIgE values, whereas normalized values were used exclusively for clustering analyses. UMAP visualization was performed on the same 15-PC subspace.

All analyses were conducted in Python 3.11 using scikit-learn, NumPy, and Matplotlib (Claude Sonnet 4.6, Anthropic, San Francisco, CA, USA).

Additional details on the computational method used are provided in Supplementary Document S1.

##### Clustering of Sensitizations to Plant Allergens

In a second phase, to better detect subtler plant-sensitization clusters that would not be apparent when all allergens were included in the PAX, we repeated the k-means analysis, restricted to the 109 pollen and plant-food (PPF) allergen extracts and components. For this sub-analysis, a dog was considered PPF-sensitized if at least one of its PPF-allergen sIgE values was 30 ng/mL or higher, as done in the full-panel clustering.

All PPF sIgE results from 6025 sensitized dogs that met the threshold were normalized per dog using the same pipeline as the full-panel analysis. Clusters were tested for k values from 5 to 15. For k = 9 to 14, a plateau was observed (scores 0.163–0.175), with no statistically meaningful optimum in this range. Accordingly, k = 10 was retained for biological comparability with the full-panel analysis and to identify rare sensitization patterns.

A post hoc analysis showed that two of the ten clusters were influenced solely by Par j 2 sIgE, differing only in their moderate versus high sIgE concentrations. Because these subclusters were qualitatively similar and merging them slightly improved the mean silhouette coefficient, they were combined into a single cluster, resulting in an effective k = 9.

Cluster characterization, allergen marker designation based on fold changes, and UMAP and heatmap visualizations were otherwise performed as described for the full-panel analysis.

## 3. Results

### 3.1. Sensitization Prevalence

Altogether, 19,151/21,121 dogs (90.7%) had at least one positive allergen in the PAX. For dogs with at least one positive test to environmental (non-insect venom) and food allergens, the numbers were 18,201 (86.2%) and 8006 (37.9%), respectively.

The sensitization prevalence (i.e., seropositivity rates) for all individual allergens tested in the PAX is reported in Supplementary Table S2, with the 20 most prevalent sensitizations listed in Table 1; the prevalence of sensitization by allergen category is shown in Table 2.

Altogether, sensitization to mites (storage and house dust mites combined) was the most prevalent category, with nearly four of five dogs sensitized to at least one mite allergen extract or component (n = 16,780/21,121; 79.5%). Among individual mite allergens, sensitization to the whole extract of *Dermatophagoides farinae* (Der f) was by far the most prevalent, with a seropositivity rate of 76.7%.

Sensitizations to insect venoms ranked second overall at 39.8%, driven primarily by those to the main allergenic venom components of honeybees (*Apis mellifera*; Api m 1, 15.6%) and wasps (*Vespula vulgaris*; Ves v 5, 13.8%). Altogether, 24.4% of dogs were sensitized to at least one honeybee allergen, 18.4% to at least one wasp allergen, and 35.4% to one or more of the Api m and Ves v allergens.

Among other environmental allergen categories, sensitizations to weed (21.5%) and tree pollens (18.6%) exceeded those to the major flea saliva allergen (Cte f 1, 7.4%), grass pollens (6.9%), animal epithelia (6.7%), molds (6.5%), *Malassezia* yeast (6.2%), and cockroaches (4.8%).

The most common sensitization to a single pollen molecular allergen was to Par j 2, the primary marker of *Parietaria* allergy in humans (10.0%), whereas the most reactive mite component was Tyr p 2 (7.2%).

Sensitization to meats was the most common food allergen category, accounting for 21.9% of dogs, mainly because of bovine serum albumin (Bos d 6; 13.2%). Less frequent sensitizations involved allergens from legumes (11.5%), eggs and milk (6.5%), cereals and grains (6.2%), fruits (6.2%), and fish (5.4%). Tubers had the lowest sensitization rate at 1.4%.

### 3.2. Unsupervised Clustering of IgE Profiles—All Allergens

#### 3.2.1. Overview of Sensitization Clusters

Among 21,121 US dogs tested over two calendar years, 16,366 (77.5%) were sensitized to at least one allergen at the 30 ng/mL sIgE threshold. K-means clustering with k = 10, selected based on clustering metrics and biological interpretability, identified four large, heterogeneous clusters (C1–C4; together comprising 86.4% of sensitized dogs) and six biologically relevant yet smaller clusters (C5–C10; together comprising 13.6%). The UMAP embedding (Supplementary Figure S1A) showed that the biologically defined clusters formed discrete islands at the centers of the four large, polysensitized groups.

#### 3.2.2. Characteristic Features of Individual Clusters

##### Heterogeneous Clusters (C1–C4)

**Figure 1:**
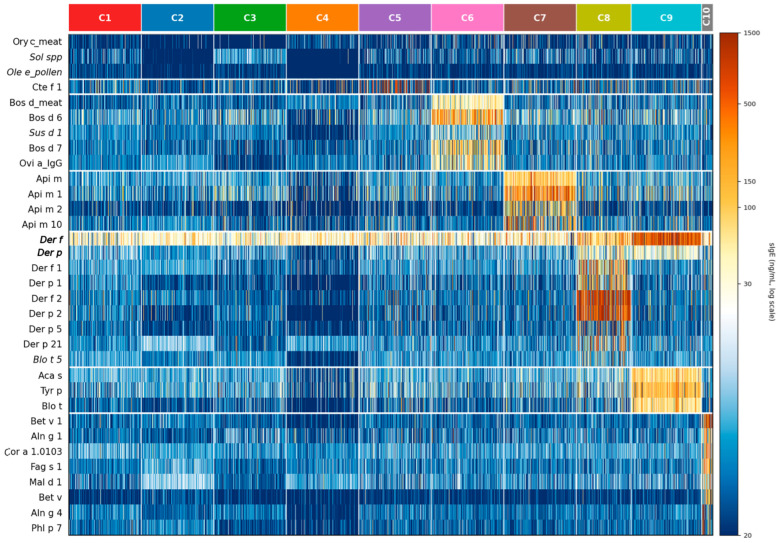
Heatmap of 16,366 sensitized dogs to all PAX allergens in the US in 2024 and 2025. Up to 350 dogs per cluster are shown, highlighting the most representative sensitization patterns. K-means clustering with k = 10 separated sensitized dogs into four large, heterogeneous (i.e., polysensitized) clusters (C1–C4) and six smaller yet biologically relevant clusters (C5–C10). Italicized allergens are supporting allergens. The cross-reactive allergen extracts of Der f and Der p (in bold italics in the heatmap) were not included in the clustering analysis due to the high prevalence of sensitization to these mites (especially Der f), but they were still added in this heatmap for biological relevance.

Clusters C1 (33.2%), C2 (29.6%), C3 (13.5%), and C4 (10.1%) had no allergens that reached the main fold threshold of 1.20 or higher (Supplementary Table S3; Figure 1), except for C4, which showed only a borderline enrichment for Ory c_meat (rabbit meat; fold 1.21×). Most sensitized dogs thus exhibited complex polysensitization patterns that did not segregate into a single dominant molecular signature. They were added to the heatmap as background context (Figure 1).

##### Flea Cluster (C5)

Cluster C5 comprised 666 dogs (4.1%) and was defined by strong enrichment of Cte f 1, the major salivary allergen of the cat flea *Ctenocephalides felis* (1.37×; Supplementary Table S3; Figure 1). There was also a minor borderline co-enrichment for Par j 2 (1.20×), which did not warrant its own cluster.

##### Serum Albumin/IgG Cross-Reactive Cluster (C6)

Cluster C6 (356 dogs, 2.2%) was defined by co-enrichment of bovine serum albumin (Bos d 6, fold 1.51×; Supplementary Table S3; Figure 1), bovine IgG (Bos d 7, 1.36×), ovine IgG (Ovi a_IgG, 1.25×), and beef meat (Bos d_meat, 1.24×), with additional support from rabbit meat (Ory c_meat, 1.12×) and porcine serum albumin (Sus d 1, 1.10×). This pattern is consistent with sensitization to mammalian serum albumins and IgGs, which are known to cross-react broadly across species.

##### Honeybee Venom Cluster (C7)

Cluster C7 (548 dogs, 3.3%) showed selective enrichment for the bee venom phospholipase A2 (Api m 1, 1.65×; Supplementary Table S3; Figure 1), the whole-bee venom extract (Api m, 1.41×), and two additional venom components (Api m 10, 1.35×; Api m 2, 1.34×). This cluster is consistent with dogs previously exposed to honeybee stings that subsequently developed sensitization to injected venom allergens.

##### House Dust Mite Group 2 Cluster (C8)

Cluster C8 (266 dogs, 1.6%) was dominated by group 2 allergens from both *Dermatophagoides* species: Der p 2 (1.98×; Supplementary Table S3; Figure 1) and Der f 2 (1.96×). There was also co-enrichment of components from HDM group 1 (Der f 1, 1.38×; Der p 1, 1.36×) and group 5/21 (Der p 5, 1.27×; Der p 21, 1.32×). Although the Der f and Der p extracts were not used in this unsupervised clustering, dogs in C8 exhibited high sIgE levels against these two extracts (Figure 1), levels surpassed only by those of dogs in the next cluster, C9. Cluster C8, centered on the main *Dermatophagoides* allergen components for humans, represents a subpopulation of dogs with distinctive sensitization to HDM molecular components.

##### Storage Mite Cluster (C9)

Cluster C9 (339 dogs, 2.1%) was co-enriched with extracts from *Tyrophagus putrescentiae* (Tyr p, 1.54×), *Blomia tropicalis* (Blo t, 1.38×), and *Acarus siro* (Aca s, 1.37×) (Supplementary Table S3; Figure 1). This triad shows a storage-mite sensitization pattern distinct from that to *Dermatophagoides* allergens and may reflect exposure to stored food products or other environments in which storage mites are abundant.

##### PR-10/Polcalcin Cluster (C10)

Cluster C10 was the smallest cluster (53 dogs, 0.3%) and showed broad enrichment across the PR-10/Bet v 1 protein family: Bet v 1 (1.71×), Aln g 1 (1.42×), Cor a 1.0103 (1.39×), Fag s 1 (1.38×), Mal d 1 (1.31×), and the whole extract of birch pollen (Bet v, 1.29×) (Supplementary Table S3; Figure 1). At this k value, canonical PR-10 allergens co-clustered with two polcalcins (Aln g 4, 1.47×; Phl p 7, 1.42×). This cluster likely represents dogs with genuine Fagales (birch-type) pollen sensitization, which is rarer in the US population than in European cohorts [9]. Importantly, PR-10 allergens from pollen co-clustered with the corresponding allergen from apples, consistent with the molecular sensitization pattern that underlies the main pollen-food allergy syndrome in humans.

### 3.3. Unsupervised Clustering of IgE Profiles—Pollen and Plant-Food Allergens

#### 3.3.1. Overview of Sensitization Clusters

**Figure 2:**
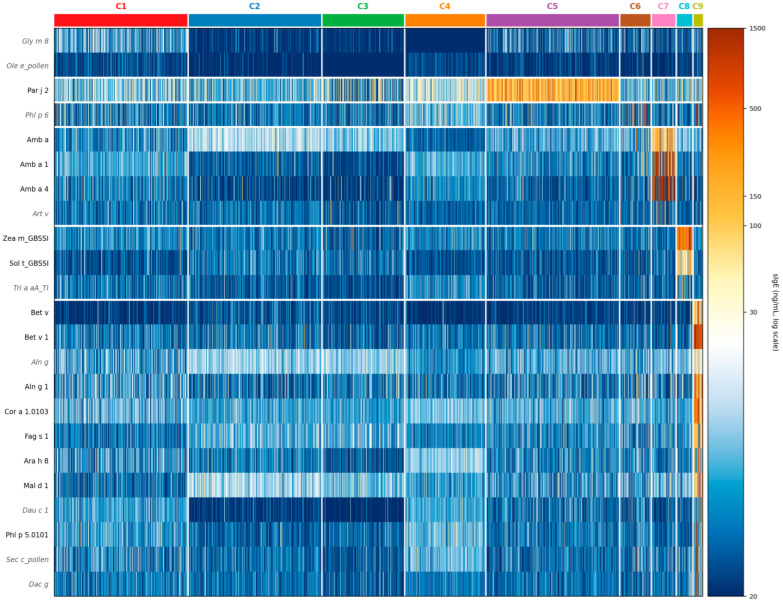
Heatmap of 6025 sensitized dogs to pollen and plant-food (PPF) allergens in the US in 2024 and 2025. Up to 350 dogs per cluster are shown, highlighting the most representative sensitization patterns. K-means clustering with k = 10, later reduced to 9 after merging two Par j 2-dominated clusters, separated sensitized dogs into four large, heterogeneous (i.e., polysensitized) clusters (C1–C4) and five smaller, biologically relevant clusters (C5–C9). Italicized allergens are supporting allergens.

Of the 21,121 dogs in the combined 2024–2025 dataset, 6025 (28.5%) were sensitized to at least one PPF allergen at the 30 ng/mL threshold. Following k-means clustering with k = 10 and post hoc merging of two quantitatively distinct Par j 2-dominated subclusters that differed only in their sIgE levels to that allergen, nine groups with distinct sIgE profiles remained (Supplementary Figure S1B; Supplementary Table S4; Figure 2). Four clusters (C1–C4, collectively accounting for 83.5% of PPF-sensitized dogs) lacked a dominant allergen (fold ≥ 1.20 across all features) and were classified as heterogeneous. The remaining five clusters (C5–C9; collectively 16.5%) each comprised between 0.4% and 12.9% of sensitized dogs and displayed well-defined allergen enrichment patterns allowing biological labels to be assigned. These clusters were centered on the Par j 2 nonspecific lipid transfer protein (nsLTP) (C5), a grass pollen component (C6), ragweed pollen (C7), GBSSI starch synthases (C8), and the PR-10 allergen family (C9).

#### 3.3.2. Characteristic Features of Individual Clusters C1–C9

##### Heterogeneous Clusters (C1–C4)

Clusters C1 (38.4%; 2313 dogs), C2 (38.0%; 2289), C3 (3.6%; 217), and C4 (3.5%; 212) together accounted for 83.5% of PPF-sensitized dogs. None of these four clusters contained allergens with a fold enrichment greater than 1.20. The only supporting allergens in C1 were Gly m 8 (soybean 2S albumin, 1.11×) and Ole e_pollen (olive tree pollen extract, 1.10×), both below the main threshold. Collectively, as seen with the unsupervised clustering of the entire panel, most dogs appear polysensitized to multiple allergens, without a dominant shared molecular recognition pattern.

##### Par j 2 Cluster (C5)

Cluster C5 (776 dogs, 12.9%) was the largest biologically defined group, characterized by elevated sIgE against Par j 2 (1.56×), a lipid transfer protein (LTP) from *Parietaria judaica* (wall pellitory) (Supplementary Table S4; Figure 2). This cluster resulted from the post hoc merging of two raw k-means clusters (raw k-means folds for Par j 2: 1.94× and 1.40×, respectively), denoting a biological continuum of Par j 2 sIgE (moderate vs. high levels) within a single underlying sensitization pattern. Because these subclusters were qualitatively indistinguishable and merging them improved the overall silhouette coefficient, they were combined into a single biological entity.

##### Phl p 6 Grass Sensitization Cluster (C6)

Cluster 6 (83 dogs, 1.4%) showed a single supporting-level enrichment for Phl p 6 (Timothy grass minor allergen; 1.15×), below the main threshold (≥1.20) (Supplementary Table S4; Figure 2). Because no allergen met the main enrichment criterion and the sole signal was a minor grass allergen component, C6 was not assigned a definitive biological label. This cluster may reflect a subpopulation with moderate, broad grass pollen sensitization, which lacks a dominant molecular driver distinct from the background clusters. Alternatively, it may represent a subpopulation of dogs sensitized only to this minor grass allergen.

##### Ragweed Cluster (C7)

Cluster 7 (65 dogs; 1.1%) showed IgE enrichment for several allergens from short, common ragweed (*Ambrosia artemisiifolia*): Amb a 4 (defensin; 1.76×), Amb a 1 (pectate lyase; 1.62×), and the Amb a whole extract (1.43×). The mugwort whole extract (*Artemisia vulgaris*, Art v; 1.13×) served as a minor supporting marker (Supplementary Table S4; Figure 2). Notably, Amb a 4 had the highest fold enrichment and prevalence in this cluster, exceeding those of Amb a 1. The Art v supporting signal is consistent with known *Ambrosia–Artemisia* cross-reactivity mediated by common pectate lyases and defensins, as well as by plant panallergens (profilins, polcalcins, and nsLTPs).

##### GBSSI Starch-Synthase Cluster (C8)

Cluster 8 (44 dogs; 0.7%) is a small cluster characterized by IgE enrichment for granule-bound starch synthases (GBSSI) from two phylogenetically distant plant families: Zea m_GBSSI (corn, Poaceae; 1.92×) and Sol t_GBSSI (potato, Solanaceae; 1.39×). The wheat alpha-amylase trypsin inhibitor (Tri a aA_TI; 1.11×) serves as a supporting marker (Supplementary Table S4; Figure 2). Among the main allergens in the PPF sub-analysis, Zea m_GBSSI showed the highest fold enrichment, highlighting the biological coherence of this cluster despite its small size. This cross-reactive sensitization pattern, spanning dicot tubers and monocot cereals, reflects the structural conservation of GBSSI across vascular plant lineages, a finding we reported previously [16].

##### PR-10 Cluster (C9)

Cluster 9 (26 dogs; 0.4%) was the smallest cluster, defined by broad IgE reactivity spanning the PR-10 homolog axis: Bet v 1 (birch; 2.12×), Aln g 1 (alder; 1.74×), Cor a 1.0103 (hazel; 1.68×), Bet v whole extract (1.51×), Fag s 1 (beech; 1.50×), Mal d 1 (apple; 1.50×), Ara h 8 (peanut; 1.28×), and the Timothy grass allergen Phl p 5.0101 (1.28×). Supporting markers included the PR-10 family allergen Dau c 1 from carrots (1.17×) and extracts from alder, rye, and orchard grass pollens (Supplementary Table S4; Figure 2). Dogs sensitized to PR-10 family allergens were already identified in the entire allergen panel clustering (cluster C10; see Section PR-10/Polcalcin Cluster (C10) above), with polcalcins also identified as main allergens of that cluster. When restricting the clustering to PPF allergens, however, polcalcins were no longer classified as main or supporting allergens; instead, extracts from alder and several grass pollen extracts or components were recognized. Altogether, these clusters highlight the common co-sensitization to PR-10 family and other pollen allergens.

## 4. Discussion

This paper reports the prevalence and profiling of sensitizations among dogs suspected of having allergies in the US in 2024 and 2025. The nearly five million individual allergen-specific IgE concentrations from more than 20,000 dogs analyzed herein largely surpass the largest sensitization survey previously published in that country [13]. Moreover, the use of datasets obtained on the same platform (PAX multiplex microarray) allows, for the first time, comparison of large-scale sensitization results from dogs across two continents (the US and Europe) [11].

Because the latest international guidelines recommend determining allergen sensitization only after the diagnosis of AD—or another allergic disease—is confirmed, it is reasonable to expect that many, and likely most, dogs tested with the PAX had a confirmed allergic disease, although this could not be verified from the laboratory database [3]. Consequently, it is not surprising that 9 of 10 dogs with allergies tested in this study had detectable sIgE against one or more allergens. It is worth noting that the prevalence of dogs with a positive PAX to at least one environmental allergen (excluding insect venoms), at 86.2%, is nearly identical to that reported by Botoni et al. (85.4%) for American dogs testing positive to any allergen by IDT and IST [17]. That about 15% of allergy-suspected dogs have a negative IgE sensitization test could be due to multiple factors, including interference from long-term administration of anti-allergic drugs with immunosuppressive effects, the seasonality of testing, the presence of low levels but high-affinity sIgE, or dogs suffering from the so-called “atopic-like dermatitis,” an entity corresponding to “intrinsic AD” in humans, in whom sIgE are largely undetectable in the skin or serum of patients [18].

While sensitization rates to most allergens in the PAX merit discussion, space limitations restrict our comments to a few notable allergens or allergen groups: mites, Hymenoptera venoms, Par j 2, and Bos d 6/Bos d 7.

Nearly 80% of allergy-suspected dogs in the US were sensitized to mites, with the *Dermatophagoides farinae* HDM (Der f; 76.7%) the most common, and the storage mite *Tyrophagus putrescentiae* (Tyr p; 12.8%) a distant second. These frequencies are similar to those reported previously in European dogs tested with PAX [11]. As observed in Europe, there was a marked discrepancy between sensitization to the Der f extract and to its major molecular allergen, Der f 2 (4.2%), whereas the difference between sensitization to the *D. pteronyssinus* HDM extract (Der p; 5.6%) and to Der p 2 (3.9%) was much smaller. These observations may reflect either that relevant Der f allergens are absent from the PAX or that a substantial proportion of reactivity to the Der f extract results from glycan-mediated cross-reactivity between high-molecular-weight Der f allergens (e.g., Der f 15, Der f 18, Zen-1) and complex carbohydrates on mucins secreted by larvae of the ubiquitous nematode *Toxocara canis*, a phenomenon which we reported recently [19]. Supporting this hypothesis is the very low prevalence of reactivity to the recombinant Der f 15 and Der f 18 allergens included in the PAX (both < 1%), which lack their natural glycans. The clinical relevance of isolated sensitization to the Der f extract in the absence of component reactivity remains unknown.

The second most prevalent category of sensitizations in this population of allergy-suspected dogs was insect venoms, with approximately 40% of dogs exhibiting IgE to one or more Hymenoptera allergens. Sensitization rates to Api m 1 and Ves v 5 exceeded those observed with the corresponding whole-venom extracts, underscoring the value of molecular allergens in detecting venom sensitization [20]. To our knowledge, this is the first report of sensitization rates to bee, wasp, and other insect venoms in dogs in the US. Although lower than those reported in European dogs [11], these frequencies indicate that exposure and sensitization to Hymenoptera venoms are common in dogs. As in humans, sensitization likely reflects prior insect stings, whereas clinical reactions probably occur only in a subset of sensitized individuals [21]. In dogs with systemic reactions following bee or wasp stings, component-resolved testing with the PAX has shown an excellent ability in identifying the culprit insect [22].

In the US, as in Europe [11], the most prevalent pollen sensitizations were directed against short ragweed (*Ambrosia artemisiifolia*) and the *Parietaria judaica* nsLTP Par j 2. While ragweed sensitization has been recognized in American dogs since the earliest IgE serological surveys [23], reports of *Parietaria* sensitization in dogs have so far been largely confined to Europe [11,24,25]. Because *P. judaica* is restricted mainly to parts of the US West Coast, the unexpectedly high prevalence of Par j 2 sensitization in our cohort (10%) is unlikely to be explained solely by exposure to this species. Instead, it more plausibly reflects sensitization to native North American *Parietaria* species, including *P. pensylvanica*, *P. floridana*, *P. hespera*, and *P. praetermissa* [26]. This interpretation is supported by the broad geographic overlap between Par j 2-positive samples and the reported distributions of several of these species (Supplementary Figure S2). Furthermore, unlike many other nsLTPs, Parj 2 shows limited cross-reactivity with homologous allergens from plants of other genera [27] and is therefore considered a marker of genuine *Parietaria* sensitization in humans [28]. Collectively, these observations suggest that exposure to North American *Parietaria* species is an underrecognized source of allergen sensitization in dogs in this country and support the inclusion of *Parietaria* extracts in intradermal and IgE serological testing panels.

In this broad dataset, a little over one-third of dogs had detectable IgE against at least one food allergen, with meats being the most common category. Among these, bovine serum albumin (Bos d 6; 13.2%) and bovine IgG (Bos d 7; 5.0%) were particularly prevalent. Although these two allergens are primarily considered serum proteins, they are commonly found in beef preparations due to residual blood and tissue fluids and are therefore relevant as beef meat allergens. Interestingly, these two proteins were the first allergens identified in dogs with food allergy reacting to beef, milk, and lamb [29,30]. In 2002, HogenEsch et al. demonstrated that vaccination, particularly with rabies vaccines, induced IgE against Bos d 6 and bovine fibronectin in laboratory dogs, with antibody levels remaining detectable for several months [31]. These proteins were presumed to originate from tissue culture media used during vaccine production and/or from stabilizers present in vaccines. Subsequent studies implicated Bos d 6 as a potential allergen involved in immediate vaccine reactions in dogs in Japan [32,33]. Because bovine IgG is also a constituent of fetal bovine serum used in cell culture systems, vaccination could, at least theoretically, also induce sensitization to Bos d 7, although direct evidence for this mechanism is currently lacking. Future studies should investigate how dietary exposure and vaccination history each contribute to sensitization against bovine serum proteins, and whether these sensitizations are associated with clinically relevant allergic reactions to vaccines, cow’s milk, beef, and other cross-reactive mammalian meats.

This is the first report of unsupervised clustering of allergen sensitization in a very large canine population. The principal finding of this analysis was that approximately 85% of sensitized dogs did not cluster into a well-defined molecular endotype but instead fell into four large clusters characterized by broad polysensitization and heterogeneous sensitization profiles. In contrast, the remaining 15% of dogs formed biologically coherent clusters dominated by sensitizations to flea saliva (C5), serum albumins and IgG (C6), honeybee venom (C7), HDM components (C8), storage mites (C9), and PR-10 proteins/polcalcins (C10). The emergence of these recognizable allergen groupings, many of which correspond to established allergen families or exposure sources, supports the biological plausibility of the molecular sensitization profiles generated by the PAX test.

One interesting feature of the clustering heatmap was the ubiquitous sensitization to the Der f extract, with low positive sIgE levels detected across all clusters, and higher concentrations in the HDM (C8) and storage mite (C9) clusters. In contrast, strong sensitization to Der p was largely restricted to these two mite-dominated clusters. This widespread distribution of Der f sensitization is consistent with our recent hypothesis that a substantial proportion of reactivity to the Der f extract reflects glycan-mediated cross-reactivity with *Toxocara canis* larvae, rather than “true primary” HDM sensitization [19]. If confirmed, this observation would further call into question the clinical relevance of isolated sensitization to the Der f extract in the absence of reactivity to *Dermatophagoides* molecular allergens.

Our clustering results differed from those reported in a recent clustering analysis of 1462 Luxembourg patients with physician-diagnosed allergies [4]. In that study, sensitized individuals segregated into seven well-defined molecular clusters, with only one cluster (C0; 24.4% of patients) showing sensitization to multiple allergen families. By comparison, our data suggest that broad polysensitization may be substantially more common in dogs than in humans with allergies. Whether this difference reflects biological distinctions between canine and human allergy, greater environmental heterogeneity within the American canine population, or a greater contribution of cross-reactive sensitizations remains unknown. Nevertheless, some similarities were apparent between the two species. Both humans and dogs exhibited clusters enriched for HDM molecular components, particularly group 2 allergens and PR-10 proteins, corresponding to the human C3 and C0 clusters and the canine C8 and C10 clusters, respectively. In contrast, the distinctive clusters of grass pollen, animal dander, and wasp venom observed in humans were not identified in this canine population.

Among the canine clusters, the serum albumin/IgG protein cluster (C6) and the PR-10/polcalcin cluster (C10) are of particular interest. The former supports the existence of a coherent sensitization pattern directed against cross-reactive mammalian serum proteins, including Bos d 6, Bos d 7, and related allergens. The latter replicates the PR-10 sensitizations observed in humans and suggests that similar pollen-associated sensitization pathways likely occur in dogs as well. Whether these molecular patterns correspond to clinically distinct phenotypes remains to be determined. In particular, it will be important to assess whether dogs within the PR-10 sensitization cluster exhibit clinical manifestations analogous to pollen-food allergy syndrome in humans [34], a condition that has thus far been documented only in one dog [35].

To identify additional pollen-associated sensitization clusters, which were more apparent in human sensitization profiles [4], we repeated the clustering using only the 109 pollen and plant food allergens. This second analysis confirmed that approximately four of five dogs were polysensitized and lacked a dominant pollen-associated molecular profile. The remaining dogs were grouped into four biologically relevant sensitization clusters to *Parietaria* (C5), ragweed (C7), granule-bound starch synthases (C8), and PR-10 family allergens (C9); one very small cluster was enriched in the Timothy grass Phl p 6 as a supporting allergen (C6).

Beyond the PR-10 allergen sensitization cluster identified in the previous analysis and its possible clinical implications, this second clustering confirmed the uniqueness of sensitization to Par j 2, further supporting the inclusion of *Parietaria* extracts in sensitization test panels in the US. The ragweed cluster was expected, given the ubiquitous distribution of this weed in North America and the strong allergenicity of some of its proteins [28]. The small cluster centered on the GBSSI protein family shows strong cross-reactivity among homologs across several plant species, as we previously suggested [16]. Whether this molecular sensitization pattern has clinical relevance is not known. If these sensitizations were to prove clinically meaningful, they could complicate the selection of starch-containing ingredients for elimination diets used during the diagnostic evaluation of food allergy.

This second analysis showed that restricting clustering to pollen and plant-food allergens revealed sensitization patterns that were not apparent in the full-allergen analysis, suggesting that dominant sensitizations to mites and other allergen sources may mask more subtle pollen-associated molecular profiles. Together, our results indicate that distinct molecular profiles of pollen-associated sensitization can still be identified within more common polysensitization repertoires. The recurrence of a PR-10 cluster across both clustering approaches further supports the existence of a coherent pollen-associated sensitization pathway in dogs.

Taken together, the predominance of heterogeneous clusters across both clustering approaches suggests that, unlike human allergy, where distinct molecular endotypes are often observed, molecular allergen sensitization in dogs is characterized predominantly by broad polysensitization and cross-reactivity rather than by a limited number of dominant molecular sensitization profiles. The relative contributions of environmental exposure, biological cross-reactivity, and other factors remain to be determined.

This study has several limitations. First, because this analysis was based on de-identified routine diagnostic submissions, standardized information on breed, age, sex, geographic location, clinical diagnosis, disease severity, treatment history, and response to therapy was not available and therefore could not be incorporated into the analyses. Consequently, the molecular sensitization profiles identified in this study should not be interpreted as clinical phenotypes or disease endotypes. Future prospective studies integrating detailed clinical metadata with molecular sensitization profiles will be needed to determine whether these clusters are associated with specific allergic diseases, patient characteristics, environmental exposures, or treatment outcomes. Second, because unique patient identifiers were unavailable, a small number of repeat submissions from individual dogs could not be excluded, but their influence is likely minimal, if any, given the size of the population analyzed. Third, allergen-specific IgE sensitization should not be equated with clinical allergy, which requires interpretation of the patient’s history, clinical signs, and diagnostic evaluation by the attending veterinarian. Finally, the cross-sectional nature of the dataset precludes assessment of longitudinal changes in sensitization within individual dogs, as shown in a recent small cohort study [36].

## 5. Conclusions

This study provides a comprehensive characterization of allergen sensitization patterns in a large cohort of American dogs with suspected allergic disease based on an analysis of more than 21,000 PAX test results. Sensitization to the house dust mite *Dermatophagoides farinae* was by far the most prevalent. However, the marked discrepancy between reactivity to the Der f extract and to its molecular allergens raises questions regarding the clinical significance of isolated Der f sensitization. In addition, sensitization to Hymenoptera venoms was common, affecting approximately 40% of tested dogs and highlighting an underappreciated source of allergen exposure in companion animals. Among pollen allergens, the frequent detection of IgE against Par j 2 revealed a previously underrecognized *Parietaria*-associated sensitization signal in American dogs with suspected allergic disease and supports the inclusion of *Parietaria* extracts in sensitization testing panels.

Our unsupervised clustering analyses showed that although most dogs with suspected allergic disease exhibit broad polysensitization rather than a limited number of dominant molecular sensitization patterns, biologically coherent sensitization signatures can still be identified. Overall, this study establishes a reference framework for molecular sensitization patterns in a large population of dogs with suspected allergic disease. Whether these molecular sensitization profiles correspond to clinically meaningful phenotypes remains an important question for future prospective studies combining standardized clinical characterization with component-resolved allergen testing. Finally, these findings provide a foundation for additional studies investigating the geographic distribution and longitudinal evolution of molecular sensitization patterns and, where detailed location metadata are available, their seasonal variation.

## Figures and Tables

**Table 1:** Top 20 sensitizations to individual allergens in dogs in the US in 2024–2025; n Positives: number of dogs sensitized to the allergen (threshold: 28 ng/mL); N Total: total number of dogs tested; % Prevalence: percentage of positive dogs over the combined 2024–2025 period.

	Allergen	Allergen Group	n Positives (2024–2025)	N Total (2024–2025)	% Prevalence (2024–2025)
1	Der f	Mites	16,202	21,121	76.7%
2	Api m 1	Insect venoms	3302	21,121	15.6%
3	Ves v 5	Insect venoms	2919	21,121	13.8%
4	Bos d 6	Meats	2792	21,121	13.2%
5	Tyr p	Mites	2710	21,121	12.8%
6	Pol d 5	Insect venoms	2312	21,121	11.0%
7	Api m	Insect venoms	2231	21,121	10.6%
8	Par j 2	Weed pollens	2109	21,121	10.0%
9	Api m 3	Insect Venoms	1798	21,121	8.5%
10	Aca s	Mites	1757	21,121	8.3%
11	Cte f 1	Fleas	1567	21,121	7.4%
12	Ves v 1	Insect venoms	1547	21,121	7.3%
13	Tyr p 2	Mites	1514	21,121	7.2%
14	Api m 10	Insect venoms	1323	21,121	6.3%
15	Amb a	Weed pollens	1300	21,121	6.2%
16	Ovi a_meat	Meats	1296	21,121	6.1%
17	Api m 2	Insect venoms	1243	21,121	5.9%
18	Der p	Mites	1188	21,121	5.6%
19	Pis s	Legumes	1106	21,121	5.2%
20	Blo t	Mites	1067	21,121	5.1%

**Table 2:** Prevalence of sensitization to allergen categories in dogs in the US in 2024–2025; n Allergens: number of allergens in the category; n Positives: number of dogs sensitized to the allergen (threshold: 28 ng/mL); N Total: total number of dogs tested; % Prevalence: percentage of positive dogs over the combined 2024–2025 period.

Rank	Allergen	n Allergens	n Positives (2024–2025)	N Total (2024–2025)	% Prevalence (2024–2025)
1	Mites	25	16,780	21,121	79.5%
2	Insect venoms	13	8415	21,121	39.8%
3	Meats	22	4626	21,121	21.9%
4	Weed pollens	18	4533	21,121	21.5%
5	Tree pollens	29	3934	21,121	18.6%
6	Legumes	21	2433	21,121	11.5%
7	Fleas	1	1567	21,121	7.4%
8	Grass pollens	15	1464	21,121	6.9%
9	Animal epithelia	13	1416	21,121	6.7%
10	Molds	12	1378	21,121	6.5%
11	Eggs & milk	11	1376	21,121	6.5%
12	*Malassezia* yeast	6	1319	21,121	6.2%
13	Cereals & grains	16	1304	21,121	6.2%
14	Fruits	5	1301	21,121	6.2%
15	Fish	18	1129	21,121	5.4%
16	Cockroaches	8	1016	21,121	4.8%
17	Tubers	5	287	21,121	1.4%

## Data Availability

All individual datasets are retained by Nextmune due to privacy restrictions. Requests for access to de-identified results may be directed to the corresponding author.

## Supplementary Materials

The following supporting information can be downloaded at: https://www.mdpi.com/article/10.3390/ani16152414/s1. Table S1: list of allergens included in the PAX for dogs version 24.1 in the US in 2024–2025; Document S1: supplementary computational methods for clustering analyses; Table S2: Sensitization prevalence to individual allergens in 21,121 dogs in the US in 2024–2025; Figure S1: Uniform manifold approximation and projection (UMAP) representation of clusters of sensitized dogs in the US in 2024 and 2025; Table S3: Full-panel IgE sensitization clusters in 16,366 dogs in the US in 2024–2025; Table S4: Pollen-plant food panel IgE sensitization clusters in 6025 dogs in the US in 2024–2025; Figure S2: Overlap between clinics reporting dogs with Par j 2 sensitization and the geographical distribution of the main North American *Parietaria* species.

## Abbreviations

The following abbreviations are used in this manuscript:

BH-FDR	Benjamini–Hochberg false discovery rate
CCDs	Cross-reactive carbohydrate determinants
IDT	Intradermal test
IST	IgE serological test
nsLTP	Nonspecific lipid transfer protein
PCA	Principal component analysis
PPF	Pollen-plant food
PR-10	Pathogenesis-related protein 10
sIgE	Specific IgE
UMAP	Uniform manifold approximation and projection
