# Supplementary material for: Molecular Allergen Sensitization Profiling in a Large Cohort of Dogs Suspected of Allergic Diseases in the USA: Prevalence and Unsupervised IgE Clustering (2024–2025)
Source: Animals (Basel). 2026 Aug 5;16(15):2414. doi: 10.3390/ani16152414 (PMC13465142; doi:10.3390/ani16152414)
Supplement: Supplementary file 1 [file animals-16-02414-s001.zip › animals-4431678-supplementary.pdf]

Table S1: list of allergens included in the PAX for dogs version 24.1 in the US in 2024-2025

Extract (E) or Component (C), mix (M)	Common name	Scientific name	Component	Biochemical designation
**Grass Pollens**				
E	Bermuda grass	*Cynodon dactylon*	Cyn d	
C	Bermuda grass	*Cynodon dactylon*	rCyn d 1	Beta-Expansin
E	Cocksfoot, orchard grass	*Dactylis glomerata*	Dac g	
E	Meadow fescue	*Festuca pratensis*	Fes p	
C	Perennial ryegrass	*Lolium perenne*	rLol p 1	Beta-Expansin
E	Bahia grass	*Paspalum notatum*	Pas n	
C	Timothy grass	*Phleum pratense*	rPhl p 1	Beta-Expansin
C	Timothy grass	*Phleum pratense*	rPhl p 2	Expansin
C	Timothy grass	*Phleum pratense*	rPhl p 5.0101	Grass group 5/6
C	Timothy grass	*Phleum pratense*	rPhl p 6	Grass group 5/6
C	Timothy grass	*Phleum pratense*	rPhl p 7	Polcalcin
C	Timothy grass	*Phleum pratense*	rPhl p 12	Profilin
E	Kentucky blue grass	*Poa pratensis*	Poa p	
E	Ryegrass, cultivated	*Secale cereale*	Sec c_pollen	
E	Johnson grass	*Sorghum halepense*	Sor h	
**Tree Pollens**				
E	Acacia	*Acacia mimosa*	Aca m	
E	Alder	*Alnus glutinosa*	Aln g	
C	Alder	*Alnus glutinosa*	rAln g 1	Pathogenesis-related protein (PR-10)
C	Alder	*Alnus glutinosa*	rAln g 4	Polcalcin
E	Silver birch	*Betula verrucosa*	Bet v	
C	Silver birch	*Betula verrucosa*	rBet v 1	Pathogenesis-related protein (PR-10)
C	Silver birch	*Betula verrucosa*	rBet v 2	Profilin
C	Silver birch	*Betula verrucosa*	rBet v 6	Isoflavone reductase
E	Hazel	*Corylus avellana*	Cor a_pollen	
C	Hazel	*Corylus avellana*	rCor a 1.0103	Pathogenesis-related protein (PR-10)
C	Japanese cedar	*Cryptomeria japonica*	nCry j 1	Pectate lyase
C	Arizona cypress	*Cupressus arizonica*	nCup a 1	Pectate lyase
E	Cypress	*Cupressus sempervirens*	Cup s	
C	Beech	*Fagus sylvatica*	rFag s 1	Pathogenesis-related protein (PR-10)
E	Ash	*Fraxinus excelsior*	Fra e	
C	Ash	*Fraxinus excelsior*	rFra e 1	Ole e 1-family
E	Walnut	*Juglans regia*	Jug r	
EM	Mountain cedar / red cedar	*Juniperus ashei / virginiana*	Jun a_Jun v	
E	Privet	*Ligustrum vulgare*	Lig v	
E	Mulberry tree	*Morus rubra*	Mor r	
E	Olive tree	*Olea europaea*	Ole e_pollen	
C	Olive tree	*Olea europaea*	rOle e 1	Ole e 1-family
C	Olive tree	*Olea europaea*	nOle e 7	Nonspecific lipid transfer protein (nsLTP)
C	Olive tree	*Olea europaea*	rOle e 9	1,3 beta-glucanase
C	London plane tree	*Platanus acerifolia*	rPla a 1	Plant invertase
C	London plane tree	*Platanus acerifolia*	nPla a 2	Polygalacturonase
C	London plane tree	*Platanus acerifolia*	rPla a 3	Nonspecific lipid transfer protein (nsLTP)
E	Cottonwood	*Populus nigra*	Pop n	
E	Elm	*Ulmus campestris*	Ulm c	
**Weed Pollens**				
E	Pigweed	*Amaranthus retroflexus*	Ama r	
E	Ragweed, short (common)	*Ambrosia artemisiifolia*	Amb a	
C	Ragweed, short (common)	*Ambrosia artemisiifolia*	rAmb a 1	Pectate lyase
C	Ragweed, short (common)	*Ambrosia artemisiifolia*	rAmb a 4	Defensin
E	Mugwort	*Artemisia vulgaris*	Art v	
C	Mugwort	*Artemisia vulgaris*	rArt v 1	Defensin
C	Mugwort	*Artemisia vulgaris*	rArt v 3	Nonspecific lipid transfer protein (nsLTP)
E	Lamb's quarter	*Chenopodium album*	Che a	
C	Lamb's quarter	*Chenopodium album*	rChe a 1	Ole e 1-family
C	Annual mercury	*Mercurialis annua*	rMer a 1	Profilin
E	Wall pellitory	*Parietaria judaica*	Par j	
C	Wall pellitory	*Parietaria judaica*	rPar j 2	Nonspecific lipid transfer protein (nsLTP)
E	Ribwort plantain	*Plantago lanceolata*	Pla l	
C	Ribwort plantain	*Plantago lanceolata*	rPla l 1	Ole e 1-family
EM	Dock/sorrel	*Rumex crispus / acetosella*	Rum c_Rum a	
E	Russian thistle	*Salsola kali*	Sal k	
C	Russian thistle	*Salsola kali*	rSal k 1	Pectin methylesterase
E	Nettle	*Urtica dioica*	Urt d	
**Animal Epithelia**				
C	Cattle	*Bos domesticus*	rBos d 2	Lipocalin
C	Guinea pig	*Cavia porcellus*	rCav p 1	Lipocalin
C	Horse	*Equus caballus*	rEqu c 1	Lipocalin
C	Horse	*Equus caballus*	nEqu c 3	Serum albumin
C	Horse	*Equus caballus*	rEqu c 4	Latherin
C	Cat	*Felis domesticus*	rFel d 1	Uteroglobin
C	Cat	*Felis domesticus*	rFel d 2	Serum albumin
C	Cat	*Felis domesticus*	rFel d 4	Lipocalin
C	Cat	*Felis domesticus*	rFel d 7	Lipocalin
C	Mouse	*Mus musculus*	rMus m 1	Lipocalin
C	Rabbit	*Oryctolagus cuniculus*	rOry c 1	Lipocalin
C	Rabbit	*Oryctolagus cuniculus*	rOry c 2	Lipocalin
C	Rabbit	*Oryctolagus cuniculus*	rOry c 3	Secretoglobin
**Cockroaches**				
E	German cockroach	*Blatella germanica*	Bla g	
C	German cockroach	*Blatella germanica*	rBla g 1	Cockroach group 1
C	German cockroach	*Blatella germanica*	rBla g 2	Aspartyl protease
C	German cockroach	*Blatella germanica*	rBla g 4	Calycin
C	German cockroach	*Blatella germanica*	rBla g 5	Glutathione S-transferase
C	German cockroach	*Blatella germanica*	rBla g 9	Arginine kinase
C	American cockroach	*Periplaneta americana*	rPer a 6	Troponin C
C	American cockroach	*Periplaneta americana*	rPer a 7	Tropomyosin
**Fleas**				
C	Cat flea	*Ctenocephalides felis*	rCte f 1	Unknown
**Storage and House Dust Mites**				
E	*Acarus siro*	*Acarus siro*	Aca s	
E	Blomia tropicalis	*Blomia tropicalis*	Blo t	
C	Blomia tropicalis	*Blomia tropicalis*	rBlo t 5	Mite, Group 5
C	Blomia tropicalis	*Blomia tropicalis*	rBlo t 10	Tropomyosin
C	Blomia tropicalis	*Blomia tropicalis*	rBlo t 21	Unknown
E	American house dust mite	*Dermatophagoides farinae*	Der f	
C	American house dust mite	*Dermatophagoides farinae*	rDer f 1	Cysteine protease
C	American house dust mite	*Dermatophagoides farinae*	rDer f 2	NPC2 family
C	American house dust mite	*Dermatophagoides farinae*	rDer f 15	Chitinase
C	American house dust mite	*Dermatophagoides farinae*	rDer f 18	Chitin-binding protein
E	European house dust mite	*Dermatophagoides pteronyssinus*	Der p	
C	European house dust mite	*Dermatophagoides pteronyssinus*	rDer p 1	Cysteine protease
C	European house dust mite	*Dermatophagoides pteronyssinus*	rDer p 2	NPC2 family
C	European house dust mite	*Dermatophagoides pteronyssinus*	rDer p 5	Unknown
C	European house dust mite	*Dermatophagoides pteronyssinus*	rDer p 7	Mite group 7
C	European house dust mite	*Dermatophagoides pteronyssinus*	rDer p 10	Tropomyosin
C	European house dust mite	*Dermatophagoides pteronyssinus*	rDer p 11	Myosin, heavy chain
C	European house dust mite	*Dermatophagoides pteronyssinus*	rDer p 20	Arginine kinase
C	European house dust mite	*Dermatophagoides pteronyssinus*	rDer p 21	Unknown
C	European house dust mite	*Dermatophagoides pteronyssinus*	rDer p 23	Peritrophin-like protein domain
C	Storage mite	*Glycyphagus domesticus*	rGly d 2	NPC2 family
E	Storage mite	*Lepidoglyphus destructor*	Lep d	
C	Storage mite	*Lepidoglyphus destructor*	rLep d 2	NPC2 family
E	Storage mite, mold mite	*Tyrophagus putrescentiae*	Tyr p	
C	Storage mite, mold mite	*Tyrophagus putrescentiae*	rTyr p 2	NPC2 family
**Molds**				
E	*Alternaria alternata*	*Alternaria alternata*	Alt a	
C	*Alternaria alternata*	*Alternaria alternata*	rAlt a 1	Alt a 1-family
C	*Alternaria alternata*	*Alternaria alternata*	rAlt a 6	Enolase
E	*Aspergillus fumigatus*	*Aspergillus fumigatus*	Asp f	
C	*Aspergillus fumigatus*	*Aspergillus fumigatus*	rAsp f 1	Mitogillin family
C	*Aspergillus fumigatus*	*Aspergillus fumigatus*	rAsp f 3	Peroxisomal protein
C	*Aspergillus fumigatus*	*Aspergillus fumigatus*	rAsp f 4	Unknown
C	*Aspergillus fumigatus*	*Aspergillus fumigatus*	rAsp f 6	Manganese superoxide dismutase
C	*Aspergillus fumigatus*	*Aspergillus fumigatus*	rAsp f 8	Ribosomal protein P2
E	*Cladosporium herbarum*	*Cladosporium herbarum*	Cla h	
C	*Cladosporium herbarum*	*Cladosporium herbarum*	rCla h 8	Short-chain dehydrogenase
E	*Penicilium chrysogenum*	*Penicilium chrysogenum*	Pen ch	
***Malassezia* Yeast**				
E	*Malassezia pachydermatis*	*Malassezia pachydermatis*	Mala p	

C	*Malassezia sympodialis*	*Malassezia sympodialis*	rMala s 1	Peroxisomal protein
C	*Malassezia sympodialis*	*Malassezia sympodialis*	rMala s 9	
C	*Malassezia sympodialis*	*Malassezia sympodialis*	rMala s 5	Unknown
C	*Malassezia sympodialis*	*Malassezia sympodialis*	rMala s 6	Cyclophilin
C	*Malassezia sympodialis*	*Malassezia sympodialis*	rMala s 11	Manganese superoxide dismutase
**Insect Venoms**				
E	Honey bee venom	*Apis mellifera*	Api m	
C	Honey bee venom	*Apis mellifera*	nApi m 1	Phospholipase A2
C	Honey bee venom	*Apis mellifera*	rApi m 2	Hyaluronidase
C	Honey bee venom	*Apis mellifera*	rApi m 3	Acid phosphatase
C	Honey bee venom	*Apis mellifera*	rApi m 5	Dipeptidylpeptidase IV
C	Honey bee venom	*Apis mellifera*	rApi m 10	Icarapin variant 2
E	Long-headed wasp venom	*Dolichovespula spp.*	Dol spp	
E	Paper wasp venom	*Polistes dominulus*	Pol d	
C	Paper wasp venom	*Polistes dominulus*	rPol d 5	Antigen 5
E	Fire ant venom	*Solenopsis richteri & Solenopsis invicta*	Sol spp	
C	Common wasp venom	*Vespula vulgaris*	rVes v 1	Phospholipase A2
C	Common wasp venom	*Vespula vulgaris*	rVes v 5	Antigen 5
E	Common wasp venom	*Vespula vulgaris*	Ves v	
**Cereals & Seeds**				
E	Oat	*Avena sativa*	Ave s	
E	Buckwheat	*Fagopyrum esculentum*	Fag e	
C	Buckwheat	*Fagopyrum esculentum*	nFag e 2	2S albumin
E	Sunflower seed	*Helianthus annuus*	Hel a	
E	Barley	*Hordeum vulgare*	Hor v	
E	Rice	*Oryza sativa*	Ory s	
C	Rice	*Oryza sativa*	rOry s_GLUB1	Glutelin B1
E	Millet	*Panicum miliaceum*	Pan m	
E	Rye, cultivated	*Secale cereale*	Sec c_flour	
E	Wheat	*Triticum aestivum*	Tri a	
C	Wheat	*Triticum aestivum*	rTri a 14	Nonspecific lipid transfer protein (nsLTP)
C	Wheat	*Triticum aestivum*	rTri a 19	Omega-5-gliadin
C	Wheat	*Triticum aestivum*	nTri a aA_TI	Alpha-amylase trypsin-inhibitor
E	Corn, cereal	*Zea mays*	Zea m	
C	Corn, cereal	*Zea mays*	rZea m 14	Nonspecific lipid transfer protein (nsLTP)
C	Corn, cereal	*Zea mays*	rZea m_GBSSI	Granule-bound starch synthase 1
**Fruits**				
C	Apple	*Malus domestica*	rMal d 1	Pathogenesis-related protein (PR-10)
C	Apple	*Malus domestica*	rMal d 2	Thaumatin-like protein
C	Apple	*Malus domestica*	rMal d 3	Nonspecific lipid transfer protein (nsLTP)
E	Tomato	*Solanum lycopersicum*	Sola l	
C	Tomato	*Solanum lycopersicum*	rSola l 6	Nonspecific lipid transfer protein (nsLTP)
**Legumes**				
C	Peanut	*Arachis hypogea*	nAra h 1	7/8S globulin
C	Peanut	*Arachis hypogea*	rAra h 2	2S albumin
C	Peanut	*Arachis hypogea*	nAra h 3	11S globulin
C	Peanut	*Arachis hypogea*	rAra h 5	Profilin
C	Peanut	*Arachis hypogea*	rAra h 6	2S albumin
C	Peanut	*Arachis hypogea*	rAra h 8	Pathogenesis-related protein (PR-10)
C	Peanut	*Arachis hypogea*	rAra h 9	Nonspecific lipid transfer protein (nsLTP)
C	Peanut	*Arachis hypogea*	rAra h 15	Oleosin
E	Soy	*Glycine max*	Gly m	
C	Soy	*Glycine max*	rGly m 4	Pathogenesis-related protein (PR-10)
C	Soy	*Glycine max*	nGly m 5	7/8S globulin
C	Soy	*Glycine max*	nGly m 6	11S globulin
C	Soy	*Glycine max*	rGly m 8	2S albumin
E	Lentil	*Lens culinaris*	Len c	
C	Lentil	*Lens culinaris*	rLen c 1	7/8S globulin
C	Lentil	*Lens culinaris*	nLen c 2	Seed-specific biotinylated protein
C	Lentil	*Lens culinaris*	rLen c 3	Nonspecific lipid transfer protein (nsLTP)
E	Pea	*Pisum sativum*	Pis s	
C	Pea	*Pisum sativum*	rPis s 1	7/8S globulin
C	Pea	*Pisum sativum*	rPis s 2	7/8S globulin
C	Pea	*Pisum sativum*	rPis s 3	Nonspecific lipid transfer protein (nsLTP)
**Tubers**				
E	Carrot	*Daucus carota*	Dau c	
C	Carrot	*Daucus carota*	rDau c 1	Pathogenesis-related protein (PR-10)
E	Potato	*Solanum tuberosum*	Sola t	
C	Potato	*Solanum tuberosum*	nSola t 2	Aspartic protease inhibitor
C	Potato	*Solanum tuberosum*	rSola t_GBSSI	Granule-bound starch synthase 1
**Eggs & Milk**				
E	Cow's milk	*Bos domesticus*	Bos d_milk	
C	Cow's milk	*Bos domesticus*	nBos d 4	Alpha-lactalbumin
C	Cow's milk	*Bos domesticus*	nBos d 5	Beta-lactoglobulin
C	Cow's milk	*Bos domesticus*	nBos d 8	Casein
E	Hen's egg white	*Gallus domesticus*	Gal d_white	
E	Hen's egg yolk	*Gallus domesticus*	Gal d_yolk	
C	Hen's egg white	*Gallus domesticus*	rGal d 1	Ovomucoid
C	Hen's egg white	*Gallus domesticus*	nGal d 2	Ovalbumin
C	Hen's egg white	*Gallus domesticus*	nGal d 3	Ovotransferrin
C	Hen's egg white	*Gallus domesticus*	nGal d 4	Lysozyme C
C	Hen's egg yolk	*Gallus domesticus*	nGal d 5	Serum albumin
**Meats**				
E	Beef	*Bos domesticus*	Bos d_meat	
C	Beef	*Bos domesticus*	nBos d 6	Serum albumin
C	Beef	*Bos domesticus*	nBos d 7	Immunoglobulin G
C	Beef	*Bos domesticus*	nBos d_ACTA1	Alpha actin
C	Beef	*Bos domesticus*	nBos d_LDHA	Lactate dehydrogenase A
E	Horse	*Equus caballus*	Equ c_meat	
E	Rabbit	*Oryctolagus spp.*	Ory c_meat	
C	Rabbit	*Oryctolagus cuniculus*	nOry c_PGM1	Phosphoglucomutase-1
C	Rabbit	*Oryctolagus cuniculus*	nOry c_PKM	Pyruvate kinase M
C	Rabbit	*Oryctolagus cuniculus*	nOry c_CKM	Creatine kinase M
C	Rabbit	*Oryctolagus cuniculus*	nOry c_GAPDH	GAPDH
C	Rabbit	*Oryctolagus cuniculus*	nOry c_TPI1	Triose phosphate isomerase 1
E	Lamb	*Ovis aries*	Ovi a_meat	
C	Lamb	*Ovis aries*	nOvi a_IgG	Immunoglobulin G
E	Pig	*Sus domesticus*	Sus d_meat	
C	Pig	*Sus domesticus*	rSus d 1	Serum albumin
E	Chicken	*Gallus domesticus*	Gal d_meat	
C	Chicken	*Gallus domesticus*	rGal d 7	Myosin light chain
C	Chicken	*Gallus domesticus*	rGal d 9	Beta-enolase (enolase -3)
C	Chicken	*Gallus domesticus*	rGal d_PKM	Pyruvate kinase M
E	Turkey	*Meleagris gallopavo*	Mel g	
E	Mealworm	*Tenebrio molitor*	Ten m	
**Fish**				
E	Herring, Atlantic	*Clupea harengus*	Clu h	
C	Herring, Atlantic	*Clupea harengus*	rClu h 1	Beta-parvalbumin
E	Cod, Atlantic	*Gadus morhua*	Gad m	
C	Cod, Atlantic	*Gadus morhua*	nGad m 1	Beta-parvalbumin
CM	Cod, Atlantic	*Gadus morhua*	nGad m 2+3	Aldolase/beta-enolase (enolase -3)
C	Cod, Atlantic	*Gadus morhua*	nGad m 4	Tropomyosin
E	Salmon, Atlantic	*Salmo salar*	Sal s	
C	Salmon, Atlantic	*Salmo salar*	rSal s 1	Beta-parvalbumin
C	Salmon, Atlantic	*Salmo salar*	rSal s 2	Beta-enolase (enolase -3)
C	Salmon, Atlantic	*Salmo salar*	rSal s 3	Aldolase
C	Salmon, Atlantic	*Salmo salar*	rSal s 4	Tropomyosin
C	Salmon, Atlantic	*Salmo salar*	nSal s 6	Collagen
C	Salmon, Atlantic	*Salmo salar*	rSal s 7	Creatine kinase M
C	Salmon, Atlantic	*Salmo salar*	rSal s 8	Triosephosphate isomerase 1
E	Mackerel, Atlantic	*Scomber scombrus*	Sco s	
C	Mackerel, Atlantic	*Scomber scombrus*	rSco s 1	Beta-parvalbumin
E	Tuna, yellowfin	*Thunnus albacares*	Thu a	
C	Tuna, yellowfin	*Thunnus albacares*	rThu a 1	Beta-parvalbumin

**75**	**Extracts**
**2**	**Extract mixes**
**160**	**Components**
**1**	**Component mix**
**238**	

r: recombinant component
n: native (purified) component

Document S1: supplementary computational methods for clustering analyses

Normalized allergen profiles were generated by dividing each allergen-specific IgE value by the total sIgE sum across the allergen panel for each dog, multiplying by 1,000, and applying a log1p transformation. The 80 most variable allergens were identified by computing per-allergen variance of the Seurat-normalized values across the 16,366 sensitized dogs before PCA. Non-sensitized dogs were excluded from this step to avoid zero-inflation bias, which would otherwise inflate the variance of high-prevalence allergens and distort feature selection.

Selected variables were standardized using z-score transformation before principal component analysis (PCA; scikit-learn version 1.4, 50 initial components). The first 15 PCs were retained for downstream analyses because they captured most of the structured variance, as indicated by the scree plot and cumulative explained variance.

K-means clustering was systematically evaluated for k = 2–20 and positivity thresholds of 28, 30, 32, and 35 ng/mL. At the selected threshold of 30 ng/mL, k = 10 yielded a local minimum in the Davies-Bouldin index (DB = 1.37) and a local peak in the silhouette score (0.252) within the k = 8–16 range, with silhouette scores for k = 11–15 declining to 0.144–0.166. Although lower k values (k = 2–6) produced marginally higher silhouette scores (0.299–0.311) (see figure below), they failed to resolve clinically distinct rare sensitization profiles as separate clusters. The value of k = 10 was therefore selected as the smallest value achieving both acceptable quantitative performance and full biological interpretability.

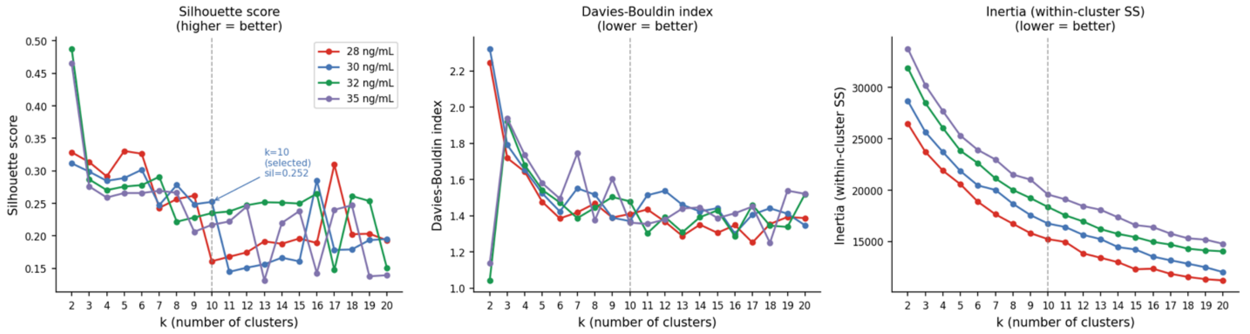

Final clustering was performed using scikit-learn KMeans with k = 10, n_init = 30, and random_state = 42. Cluster labels were assigned in descending order of cluster size. Two post hoc display-order adjustments were applied to improve the biological interpretability of visualizations: the serum albumin and honeybee venom clusters were swapped, as were the storage mite and house dust mite group 2 clusters.Fold enrichment was calculated from mean log10(sIgE + 1) values. To avoid instability due to near-zero denominators, a minimum denominator of 0.05 was applied. Allergens with fold enrichment of at least 1.20 were designated major cluster markers, whereas those with fold enrichment between 1.10 and 1.19 were designated supporting markers.

Heatmaps displayed 38 allergen rows (Der f plus 37 selected allergens) across cluster columns. Up to 350 representative dogs per cluster were sampled for visualization. The heatmap color scale used a 10-stop colormap normalized with TwoSlopeNorm (vmin = log10(21), vcenter = log10(31), vmax = log10(1501)), with the white midpoint set to the selected positivity threshold of 30 ng/mL. UMAP visualization was computed using n_neighbors = 20, min_dist = 0.3, Euclidean metric, and random_state = 42 on the same 15-PC subspace used for clustering.

Table S2: Sensitization prevalence to individual allergens in 21,121 dogs in the US in 2024-2025

Rank	Allergen	Allergen Group	n Positive (2024-2025)	N Total (2024-2025)	% Prevalence (2024-2025)
1	Der f	Mites	16 202	21 121	76,7%
2	Api m 1	Insect venoms	3 302	21 121	15,6%
3	Ves v 5	Insect venoms	2 919	21 121	13,8%
4	Bos d 6	Meats	2 792	21 121	13,2%
5	Tyr p	Mites	2 710	21 121	12,8%
6	Pol d 5	Insect venoms	2 312	21 121	11,0%
7	Api m	Insect venoms	2 231	21 121	10,6%
8	Par j 2	Weed pollens	2 109	21 121	10,0%
9	Api m 3	Insect venoms	1 798	21 121	8,5%
10	Aca s	Mites	1 757	21 121	8,3%
11	Cte f 1	Fleas	1 567	21 121	7,4%
12	Ves v 1	Insect venoms	1 547	21 121	7,3%
13	Tyr p 2	Mites	1 514	21 121	7,2%
14	Api m 10	Insect venoms	1 323	21 121	6,3%
15	Amb a	Weed pollens	1 300	21 121	6,2%
16	Ovi a_meat	Meats	1 296	21 121	6,1%
17	Api m 2	Insect venoms	1 243	21 121	5,9%
18	Der p	Mites	1 188	21 121	5,6%
19	Pis s	Legumes	1 106	21 121	5,2%
20	Blo t	Mites	1 067	21 121	5,1%
21	Bos d 7	Meats	1 053	21 121	5,0%
22	Bos d_meat	Meats	891	21 121	4,2%
23	Der f 2	Mites	887	21 121	4,2%
24	Mal d 1	Fruits	881	21 121	4,2%
25	Der p 21	Mites	856	21 121	4,1%
26	Der p 2	Mites	833	21 121	3,9%
27	Aln g	Tree pollens	718	21 121	3,4%
28	Aca m	Tree pollens	710	21 121	3,4%
29	Ovi a_IgG	Meats	669	21 121	3,2%
30	Dol spp	Insect venoms	651	21 121	3,1%
31	Der f 1	Mites	644	21 121	3,1%
32	Ves v	Insect venoms	570	21 121	2,7%
33	Der p 1	Mites	522	21 121	2,5%
34	Mala s 1	Molds	487	21 121	2,3%
35	Jun a_Jun v	Tree pollens	477	21 121	2,3%
36	Aln g 1	Tree pollens	450	21 121	2,1%
37	Fel d 2	Animal epithelia	446	21 121	2,1%
38	Ave s	Cereals & grains	429	21 121	2,0%
39	Phl p 1	Grass pollens	412	21 121	2,0%
	Ory c_meat	Meats	412	21 121	2,0%
41	Per a 7	Cockroaches	406	21 121	1,9%
42	Sus d 1	Meats	384	21 121	1,8%
43	Fag s 1	Tree pollens	375	21 121	1,8%
44	Phl p 6	Grass pollens	366	21 121	1,7%
45	Mal d 3	Fruits	346	21 121	1,6%
46	Der p 5	Mites	344	21 121	1,6%
47	Lep d	Mites	337	21 121	1,6%
48	Asp f 6	Molds	332	21 121	1,6%
49	Mala s 11	Molds	318	21 121	1,5%
50	Pla l	Weed pollens	311	21 121	1,5%
51	Bet v 6	Tree pollens	305	21 121	1,4%
52	Mala s 9	Molds	297	21 121	1,4%
53	Sal k 1	Weed pollens	296	21 121	1,4%
54	Amb a 4	Weed pollens	293	21 121	1,4%
55	Bos d 8	Eggs & milk	288	21 121	1,4%
56	Asp f 1	Molds	285	21 121	1,4%
57	Bos d_milk	Eggs & milk	281	21 121	1,3%
58	Mala p	Molds	279	21 121	1,3%
59	Ten m	Meats	269	21 121	1,3%
60	Cor a 1.0103	Tree pollens	268	21 121	1,3%
61	Gad m	Fish	266	21 121	1,3%
62	Alt a 1	Molds	264	21 121	1,3%
63	Blo t 5	Mites	257	21 121	1,2%
64	Gly m 5	Legumes	256	21 121	1,2%
65	Gly d 2	Mites	252	21 121	1,2%
	Gly m 8	Legumes	252	21 121	1,2%
67	Cry j 1	Tree pollens	250	21 121	1,2%
68	cup a 1	Tree pollens	246	21 121	1,2%
69	Gal d 2	Eggs & milk	245	21 121	1,2%
70	Cup s	Tree pollens	242	21 121	1,2%
71	Sal k	Weed pollens	241	21 121	1,1%
72	Blo t 10	Mites	239	21 121	1,1%
73	Bla g 2	Cockroaches	237	21 121	1,1%
74	Gal d_white	Eggs & milk	232	21 121	1,1%
75	Fel d 1	Animal epithelia	226	21 121	1,1%
76	Der p 10	Mites	218	21 121	1,0%
77	Bla g 5	Cockroaches	217	21 121	1,0%
78	Mala s 5	Molds	215	21 121	1,0%
79	Phl p 2	Grass pollens	206	21 121	1,0%
	Ama r	Weed pollens	206	21 121	1,0%
	Gal d 4	Eggs & milk	206	21 121	1,0%
82	Ole e 1	Tree pollens	198	21 121	0,9%
	Pol d	Insect venoms	198	21 121	0,9%
84	Der p 23	Mites	197	21 121	0,9%
85	Amb a 1	Weed pollens	195	21 121	0,9%
86	Len c	Legumes	194	21 121	0,9%
	Bet v 1	Tree pollens	193	21 121	0,9%

Rank	Allergen	Allergen Group	n Positive (2024-2025)	N Total (2024-2025)	% Prevalence (2024-2025)
120	Phl p 5.0101	Grass pollens	143	21 121	0,7%
	Ole e 7	Tree pollens	143	21 121	0,7%
122	Len c 2	Legumes	140	21 121	0,7%
123	Blo t 21	Mites	137	21 121	0,7%
124	Asp f 4	Molds	134	21 121	0,6%
125	Bla g 9	Cockroaches	132	21 121	0,6%
126	Equ c 4	Animal epithelia	130	21 121	0,6%
127	Der p 7	Mites	129	21 121	0,6%
128	Mala s 6	Molds	127	21 121	0,6%
129	Lep d 2	Mites	126	21 121	0,6%
130	Sec c_flour	Cereals & grains	124	21 121	0,6%
131	Sco s	Fish	120	21 121	0,6%
132	Cav p 1	Animal epithelia	119	21 121	0,6%
133	Fes p	Grass pollens	118	21 121	0,6%
	Hor v	Cereals & grains	118	21 121	0,6%
135	Pas n	Grass pollens	117	21 121	0,6%
	Sec c_pollen	Grass pollens	117	21 121	0,6%
137	Ole e 9	Tree pollens	116	21 121	0,6%
	Fag e	Cereals & grains	116	21 121	0,6%
139	Der f 18	Mites	114	21 121	0,5%
140	Tri a 19	Cereals & grains	112	21 121	0,5%
141	Pis s 2	Legumes	110	21 121	0,5%
142	Fel d 4	Animal epithelia	109	21 121	0,5%
143	Thu a 1	Fish	108	21 121	0,5%
144	Art v 3	Weed pollens	107	21 121	0,5%
145	Clu h 1	Fish	104	21 121	0,5%
146	Pla a 3	Tree pollens	103	21 121	0,5%
147	Ara h 1	Legumes	102	21 121	0,5%
	Gal d 3	Eggs & milk	102	21 121	0,5%
149	Mel g	Meats	95	21 121	0,5%
150	Gal d_meat	Meats	91	21 121	0,4%
151	Bla g 4	Cockroaches	88	21 121	0,4%
152	Gad m 4	Fish	87	21 121	0,4%
153	Gal d 1	Eggs & milk	86	21 121	0,4%
154	Pis s 3	Legumes	83	21 121	0,4%
155	Sol t_GBSSI	Tubers	81	21 121	0,4%
156	Phl p 12	Grass pollens	76	21 121	0,4%
157	Dau c 1	Tubers	74	21 121	0,4%
158	Ory c 1	Animal epithelia	73	21 121	0,4%
159	Asp f	Molds	72	21 121	0,3%
160	Hel a	Cereals & grains	70	21 121	0,3%
	Gal d_PKM	Meats	70	21 121	0,3%
	Sol t	Tubers	70	21 121	0,3%
163	Pan m	Cereals & grains	69	21 121	0,3%
164	Len c 1	Legumes	68	21 121	0,3%
165	Fel d 7	Animal epithelia	67	21 121	0,3%
	Sal s 7	Fish	67	21 121	0,3%
167	Bet v 2	Tree pollens	66	21 121	0,3%
	Zea m 14	Cereals & grains	66	21 121	0,3%
169	Cyn d 1	Grass pollens	65	21 121	0,3%
	Poa p	Grass pollens	65	21 121	0,3%
171	Ory s	Cereals & grains	64	21 121	0,3%
	Gal d 5	Eggs & milk	64	21 121	0,3%
173	Cor a_pollen	Tree pollens	62	21 121	0,3%
174	Zea m	Cereals & grains	61	21 121	0,3%
	Ara h 3	Legumes	61	21 121	0,3%
176	Fag e 2	Cereals & grains	59	21 121	0,3%
	Sol t 2	Tubers	59	21 121	0,3%
178	Bet v	Tree pollens	58	21 121	0,3%
	Tri a	Cereals & grains	58	21 121	0,3%
180	Sal s 4	Fish	57	21 121	0,3%
181	Pis s 1	Legumes	56	21 121	0,3%
182	Lig v	Tree pollens	55	21 121	0,3%
183	Lol p 1	Grass pollens	54	21 121	0,3%
	Gad m 2+3	Fish	54	21 121	0,3%
	Sola l 6	Fruits	54	21 121	0,3%
186	Per a 6	Cockroaches	53	21 121	0,3%
	Ara h 2	Legumes	53	21 121	0,3%
	Ara h 9	Legumes	53	21 121	0,3%
189	Che a	Weed pollens	52	21 121	0,3%
	Ory c 3	Animal epithelia	52	21 121	0,3%
	Equ c_meat	Meats	52	21 121	0,3%
192	Ulm c	Tree pollens	51	21 121	0,2%
	Ara h 5	Legumes	51	21 121	0,2%
194	Art v 1	Weed pollens	50	21 121	0,2%
195	Tri a aA_TI	Cereals & grains	49	21 121	0,2%
	Gly m 4	Legumes	49	21 121	0,2%
	Ory c_CKM	Meats	49	21 121	0,2%
198	Equ c 1	Animal epithelia	46	21 121	0,2%
199	Sal s 8	Fish	45	21 121	0,2%
200	Dac g	Grass pollens	44	21 121	0,2%
201	Ara h 15	Legumes	42	21 121	0,2%
202	Sal s	Fish	41	21 121	0,2%
203	Art v	Weed pollens	39	21 121	0,2%
	Mer a 1	Weed pollens	39	21 121	0,2%
	Gal d 9	Meats	39	21 121	0,2%
	Clu h	Fish	39	21 121	0,2%

**87**	**Pop n**	Tree pollens	193	21 121	0,9%
	**Rum a_Rum c**	Weed pollens	193	21 121	0,9%
	**Sal s 1**	Fish	193	21 121	0,9%
**91**	**Sol spp**	Insect venoms	188	21 121	0,9%
	**Zea m_GBSSI**	Cereals & grains	188	21 121	0,9%
**93**	**Alt a**	Molds	185	21 121	0,9%
**94**	**Bos d 4**	Eggs & milk	182	21 121	0,9%
**95**	**Der p 20**	Mites	179	21 121	0,9%
**96**	**Der p 11**	Mites	176	21 121	0,8%
**97**	**Cla h 8**	Molds	174	21 121	0,8%
**98**	**Aln g 4**	Tree pollens	172	21 121	0,8%
**99**	**Gly m**	Legumes	169	21 121	0,8%
**100**	**Ara h 8**	Legumes	164	21 121	0,8%
**101**	**Asp f 3**	Molds	163	21 121	0,8%
	**Sola l**	Fruits	163	21 121	0,8%
**103**	**Ory c 2**	Animal epithelia	162	21 121	0,8%
**104**	**Che a 1**	Weed pollens	161	21 121	0,8%
	**Sus d_meat**	Meats	161	21 121	0,8%
**106**	**Phl p 7**	Grass pollens	154	21 121	0,7%
	**Par j**	Weed pollens	154	21 121	0,7%
**108**	**Pla a 2**	Tree pollens	153	21 121	0,7%
	**Gal d_yolk**	Eggs & milk	153	21 121	0,7%
**110**	**Jug r_pollen**	Tree pollens	151	21 121	0,7%
**111**	**Pla l 1**	Weed pollens	150	21 121	0,7%
	**Der f 15**	Mites	150	21 121	0,7%
	**Bos d 5**	Eggs & milk	150	21 121	0,7%
	**Ory c_PGM1**	Meats	150	21 121	0,7%
**115**	**Sco s 1**	Fish	149	21 121	0,7%
	**Thu a**	Fish	149	21 121	0,7%
**117**	**Pla a 1**	Tree pollens	148	21 121	0,7%
	**Gly m 6**	Legumes	148	21 121	0,7%

**207**	**Mus m 1**	Animal epithelia	37	21 121	0,2%
**208**	**Ory s_GLUB1**	Cereals & grains	35	21 121	0,2%
**209**	**Fra e**	Tree pollens	34	21 121	0,2%
	**Fra e 1**	Tree pollens	34	21 121	0,2%
	**Bos d 2**	Animal epithelia	34	21 121	0,2%
	**Alt a 6**	Molds	34	21 121	0,2%
	**Gad m 1**	Fish	34	21 121	0,2%
**214**	**Bos d_ACTA1**	Meats	32	21 121	0,2%
	**Asp f 8**	Molds	32	21 121	0,2%
**216**	**Sor h**	Grass pollens	31	21 121	0,2%
**217**	**Len c 3**	Legumes	30	21 121	0,1%
**218**	**Bla g 1**	Cockroaches	29	21 121	0,1%
	**Tri a 14**	Cereals & grains	29	21 121	0,1%
	**Mal d 2**	Fruits	29	21 121	0,1%
	**Gal d 7**	Meats	29	21 121	0,1%
**222**	**Api m 5**	Insect venoms	26	21 121	0,1%
**223**	**Cla h**	Molds	24	21 121	0,1%
**224**	**Dau c**	Tubers	19	21 121	0,1%
**225**	**Cyn d**	Grass pollens	17	21 121	0,1%
	**Ory c_TPI1**	Meats	17	21 121	0,1%
**227**	**Urt d**	Weed pollens	15	21 121	0,1%
	**Ara h 6**	Legumes	15	21 121	0,1%
**229**	**Sal s 2**	Fish	11	21 121	0,1%
**230**	**Bos d_LDHA**	Meats	9	21 121	0,04%
**231**	**Mor r**	Tree pollens	7	21 121	0,03%
	**Ory c_PKM**	Meats	7	21 121	0,03%
	**Sal s 3**	Fish	7	21 121	0,03%
	**Bla g**	Cockroaches	7	21 121	0,03%
**235**	**Ole e_pollen**	Tree pollens	6	21 121	0,03%
	**Ory c_GAPDH**	Meats	6	21 121	0,03%
	**Sal s 6**	Fish	6	21 121	0,03%

Figure S1: Uniform manifold approximation and projection (UMAP) representation of clusters of sensitized dogs in the US in 2024 and 2025

A. Clustering of 16,366 dogs based on sensitization to all allergens

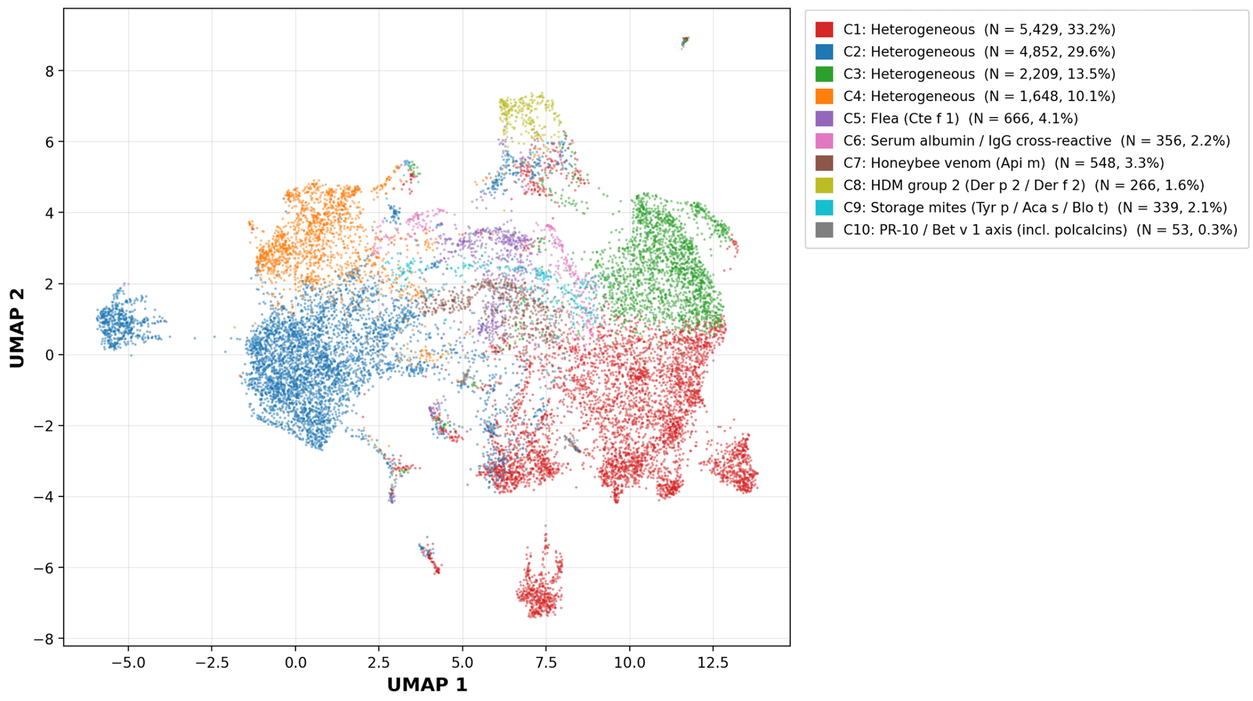

B. Clustering of 6,025 dogs based on pollen and plant food allergen sensitization

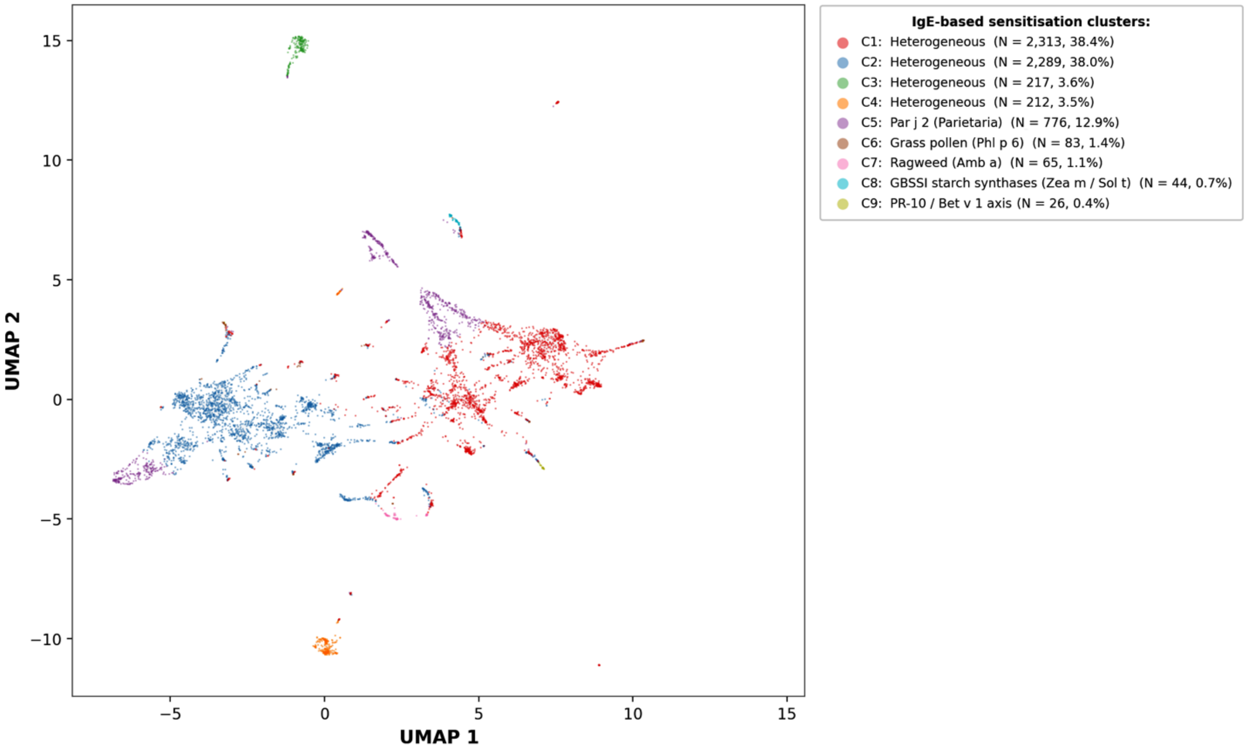

Table S3: Full-panel IgE sensitization clusters in 16,366 dogs in the US in 2024-2025

Cluster	N (dogs)	% of total	Cluster characterization	Main allergens (fold ≥ 1.20)	Supporting allergens (fold 1.10–1.19)	Notes
C1	5 429	33,2%	Heterogeneous	–	Ory c_meat (1.19×, prev. 1.8%)<br>Sol spp (1.17×, prev. 0.8%)<br>Ole e_pollen (1.11×, prev. 0.1%)	No allergen reaches fold ≥ 1.20. Broad, undifferentiated sensitization without a dominant allergen driver.
C2	4 852	29,6%	Heterogeneous	–	Blo t 10 (1.17×, prev. 0.5%)<br>Api m 3 (1.13×, prev. 10.7%)	No allergen reaches fold ≥ 1.20. Broad, undifferentiated sensitization without a dominant allergen driver.
C3	2 209	13,5%	Heterogeneous	–	Sol spp (1.16×, prev. 2.6%)	No allergen reaches fold ≥ 1.20. Broad, undifferentiated sensitization without a dominant allergen driver.
C4	1 648	10,1%	Heterogeneous	Ory c_meat (1.21×, prev. 2.5%)	Blo t 10 (1.17×, prev. 3.5%)	Only 1 allergen reaches fold ≥ 1.20. Broad, undifferentiated sensitization without a really-dominant allergen driver.
C5	666	4,1%	Flea saliva (Cte f 1)	Cte f 1 (1.37×, prev. 37.5%)<br>Par j 2 (1.20×, prev. 33.0%)	–	Flea allergen cluster. Cte f 1 (*Ctenocephalides felis*) is the major flea saliva allergen.
C6	356	2,2%	Serum albumin / IgG	Bos d 6 (1.51×, prev. 87.6%)<br>Bos d 7 (1.36×, prev. 75.0%)<br>Ovi a_IgG (1.25×, prev. 62.9%)<br>Bos d_meat (1.24×, prev. 84.0%)	Ory_meat (1.12×, prev. 13.5%)<br>Sus d 1 (1.10×, prev. 34.3%)	Cross-reactive mammalian serum albumins (Bos d 6, Sus d 1), IgGs (Bos d 7, Ovi a_IgG) and meat proteins. Consistent with mammalian meat syndrome or cross-reactive albumin/IgG sensitization.
C7	548	3,3%	Honeybee venom (Api m)	Api m 1 (1.65×, prev. 94.7%)<br>Api m (1.41×, prev. 97.4%)<br>Api m 10 (1.35×, prev. 54.0%)<br>Api m 2 (1.34×, prev. 62.0%)	–	Honeybee venom sensitization. Api m 1 (phospholipase A2) and Api m (whole venom extract) are the major allergens in this cluster.
C8	266	1,6%	HDM group 2 (Der p 2 / Der f 2)	Der p 2 (1.98×, prev. 89.1%)<br>Der f 2 (1.96×, prev. 88.3%)<br>Der f 1 (1.38×, prev. 57.9%)<br>Der p 1 (1.36×, prev. 54.1%)<br>Der p 21 (1.32×, prev. 44.4%)<br>Der p 5 (1.27×, prev. 36.5%)	Blo t 5 (1.14×, prev. 24.8%)	House dust mite cluster dominated by group 2 allergens (NPC2 lipid-binding proteins). Der p 2 and Der f 2 are the key discriminating allergens in this cluster. They are supported by HDM group 1 and group 5/21 allergens.
C9	339	2,1%	Storage mites	Tyr p (1.54×, prev. 96.5%)<br>Blo t (1.38×, prev. 94.4%)<br>Aca s (1.37×, prev. 98.5%)	–	Storage mite cluster. *Tyrophagus putrescentiae* (Tyr p) and *Acarus siro* (Aca s) are the dominant species; *Blomia tropicalis* (Blo t) co-sensitisation likely represents cross-reactivity.
C10	53	0,3%	PR-10s and polcalcins	Bet v 1 (1.71×, prev. 67.9%)<br>Aln g 4 (1.47×, prev. 43.4%)<br>Aln g 1 (1.42×, prev. 66.0%)<br>Phl p 7 (1.42×, prev. 45.3%)<br>Cor a 1.0103 (1.39×, prev. 62.3%)<br>Fag s 1 (1.38×, prev. 67.9%)<br>Mal d 1 (1.31×, prev. 54.7%)<br>Bet v (1.29×, prev. 52.8%)	Ara h 8 (1.15×, prev. 28.3%)<br>Phl p 5.0101 (1.14×, prev. 17.0%)<br>Gly m 4 (1.13×, prev. 26.4%)<br>Cor a_pollen (1.13×, prev. 35.8%)	Cluster dominated by sensitization to cross-reactive pollen-plant food allergens of the PR-10 family (Bet v 1, Aln g 1, Cor a 1, Fag s 1, Mal d 1, Ara h 8 and Gly m 4) and their extracts (Bet v, Cor a_pollen). Two polcalcins (Aln g 4, Phl p 7) also belong to this cluster.

k-means (k=10) on $\log_{10}$(sIgE+1) matrix (top-80 variable allergens, PCA) | Der f & Der p excluded from clustering features | fold enrichment = mean $\log_{10}$(sIgE+1) in cluster ÷ mean in all other clusters | prev.: prevalence

Table S4: Pollen-plant food panel IgE sensitization clusters in 6,025 dogs in the US in 2024-2025

Cluster	N (dogs)	% of total	Cluster characterization	Main allergens (fold ≥1.20)	Supporting allergens (fold 1.10–1.19)	Notes
C1	2 313	38,4%	Heterogeneous	–	Gly m 8 (1.11×, prev. 3.0%)<br>Ole e_pollen (1.10×, prev. 0.1%)	Largest cluster (38.4%); no allergen reaches fold ≥ 1.20. Gly m 8 (soybean 2S albumin) is the sole supporting signal (1.11×). Consistent with heterogeneous polysensitisation to pollen and plant foods without a dominant shared allergen recognition pattern.
C2	2 289	38,0%	Heterogeneous	–	–	Second largest cluster (38.0%); no enriched allergen above the supporting threshold. Together with C1, these two background clusters account for 76.4% of PF-sensitised dogs, reflecting the absence of a dominant molecular driver across most pollen/plant food-sensitised US dogs.
C3	217	3,6%	Heterogeneous	–	–	Third background cluster (3.6%); no enriched allergen above the supporting threshold. Consistent with heterogeneous polysensitisation without a detectable shared molecular pattern.
C4	212	3,5%	Heterogeneous	–	–	Fourth background cluster (3.5%); no enriched allergen above the supporting threshold. Similar to C3, represents broad background sensitisation without cluster-defining allergen specificity.
C5	776	12,9%	Parietaria LTP (Par j 2)	Par j 2 (1.56×, prev. 31.1%)	–	Largest signal cluster (12.9%). Par j 2 is a nonspecific lipid transfer protein (nsLTP) from *Parietaria judaica* (wall pellitory), which is a marker for primary *Parietaria* allergy in humans. *P. judaica* is largely absent in the US outside of the west coast, so sensitization to this allergen likely represents cross-reactivity with a close homolog of another *Parietaria* species, such as P. pensylvanica, floridiana, or hespera.
C6	83	1,4%	Grass pollen (Phl p 6)	–	Phl p 6 (1.15×, prev. 5.3%)	Small cluster (1.4%). Phl p 6 (Timothy grass, a minor grass allergen) does not reach the main threshold (1.15×). This cluster may reflect general grass pollen sensitisation without a single dominant molecular marker, or a subpopulation sensitised to minor grass allergens.
C7	65	1,1%	Ragweed (Amb a)	Amb a 4 (1.76×, prev. 4.5%)<br>Amb a 1 (1.62×, prev. 2.9%)<br>Amb a (1.43×, prev. 6.9%)	Art v (1.13×, prev. 0.5%; tentative)	Ragweed sensitisation cluster (1.1%). Amb a 4 (defensin-like, 1.76×) is the highest-fold allergen; Amb a 1 (pectate lyase, major allergen) and the ragweed extract (Amb a) are also enriched. Art v (mugwort) is a supporting allergen, consistent with known *Artemisia–Ambrosia* cross-reactivity via defensin, pectate lyase and other homologs. The prevalence of main allergens is low (< 7%), suggesting this is a genuinely ragweed-primary cluster rather than a broad weed sensitisation cluster.
C8	44	0,7%	Starch synthases (GBSSI)	Zea m_GBSSI (1.92×, prev. 2.7%)<br>Sol t_GBSSI (1.39×, prev. 1.0%)	Tri a aA_TI (1.11×, prev. 0.6%)	Rare cluster (0.7%). GBSSI (granule-bound starch synthase) is a conserved plant enzyme present in corn (Zea m_GBSSI, 1.92×) and potato (Sol t_GBSSI, 1.39×). The known cross-reactivity between cereal and tuber starch synthases drives co-sensitisation. Tri a aA_TI (wheat alpha-amylase/trypsin inhibitor, 1.11×) as supporting allergen suggests a possible co-sensitisation to wheat seed storage proteins.
C9	26	0,4%	PR-10 / Bet v 1 axis	Bet v 1 (2.12×, prev. 2.7%)<br>Aln g 1 (1.74×, prev. 6.0%)<br>Cor a 1.0103 (1.68×, prev. 3.0%)<br>Bet v (1.51×, prev. 0.8%)<br>Fag s 1 (1.50×, prev. 2.8%)<br>Mal d 1 (1.50×, prev. 5.1%)<br>Phl p 5.0101 (1.28×, prev. 1.8%)<br>Ara h 8 (1.28×, prev. 2.2%)	Aln g (1.18×, prev. 4.1%)<br>Dau c 1 (1.17×, prev. 1.0%)<br>Sec c_pollen (1.17×, prev. 1.6%)<br>Dac g (1.17×, prev. 0.6%)	Rarest cluster (0.4%). There is broad PR-10 sensitisation spanning birch (Bet v 1, 2.12×), alder (Aln g 1, 1.74×), hazel (Cor a 1.0103, 1.68×), beech (Fag s 1, 1.50×) and plant food PR-10 homologs (Mal d 1, apple; Ara h 8, peanut). Phl p 5.0101 (grass group 5) signals suggest co-sensitisation spanning multiple pollen protein families.

k-means (k=10) with merging of 2 Par j 2 clusters on $\log_{10}$(sIgE+1) matrix (top-80 variable allergens, PCA) | fold enrichment = mean $\log_{10}$ in cluster / mean $\log_{10}$ in all other clusters | prev. = prevalence (% of 6,025 PF-sensitised dogs ≥30 ng/mL)

Figure S2: Overlap between clinics reporting dogs with Par j 2 sensitization and the geographical distribution of the main North American *Parietaria* species

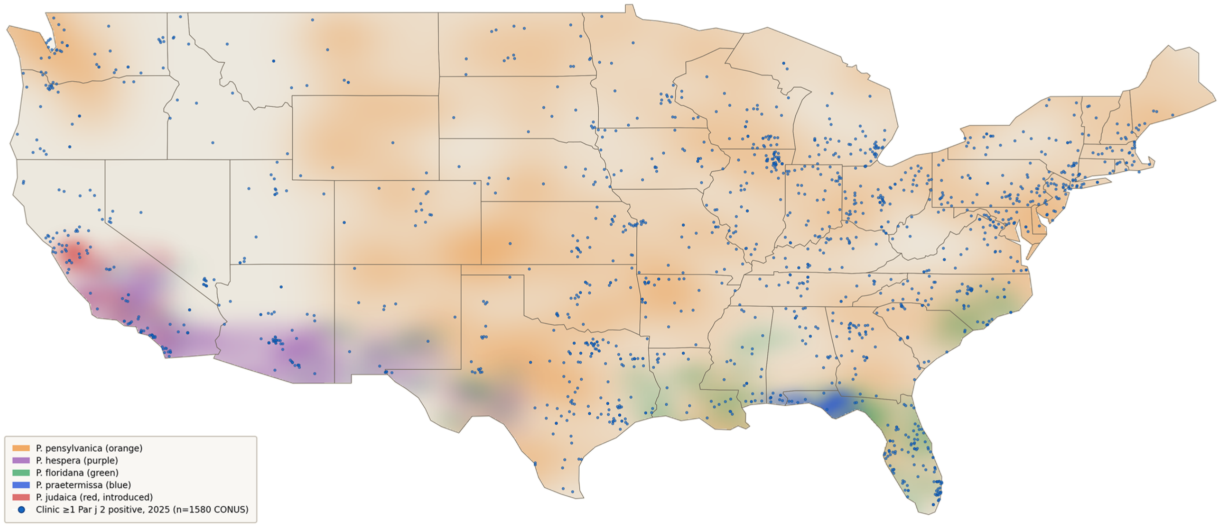

Each blue dot marks the center of a zip-code for a clinic that submitted at least one canine serum with Par j 2 sIgE of at least 28 ng/mL in 2025. The ranges of the various *Parietaria* species were assessed by consulting www.gbif.org, entering each species name followed by a date restriction from 2000 to 2026 to ensure the most current geographical distribution.
